# Anti-oncogenic and immunological functions of *ATP23* in CMS4 colon adenocarcinoma based on a machine learning computational framework

**DOI:** 10.7717/peerj.20838

**Published:** 2026-02-20

**Authors:** Yafei Yin, Huimin Zhang, Shuai Li, Ruru Gu, Jian Wang, Zhen Zhang, Juntao Sun

**Affiliations:** 1 Department of Gastroenterology, The Second Qilu Hospital of Shandong University, Jinan, China; 2 Department of Gastroenterology, Dongying District People’s Hospital, Dongying, China; 3 Department of Gastrointestinal Endoscopy Center, The Second Qilu Hospital of Shandong University, Jinan, China

**Keywords:** Colon adenocarcinoma, ATP23, Immune response, Prognostic biomarker

## Abstract

**Background:**

Consensus Molecular Subtype (CMS) 4 and BRAF mutations are poor prognostic indicators for colon adenocarcinoma (COAD). Although the prevalence of BRAF-mutated COAD is higher in the CMS1 subtype, we have identified certain cases of CMS4 subtypes in patients with BRAF mutations. However, there is currently a lack of research exploring whether this particular type of COAD exhibits a worse prognosis and unraveling its underlying mechanism.

**Methods:**

This retrospective study analyzed the transcriptome profiles and clinical parameters of COAD patients from six public datasets. Kaplan–Meier plots and bioinformatics methods predicted the correlation between ATP23 expression and patient survival. We compared enriched pathways, genomic mutations, immune cell infiltration, copy number alterations, cell–cell communication, and TIDE scores between ATP23-high and ATP23-low groups. Furthermore, *in vitro* experiments verified the potential roles of ATP23 in COAD.

**Results:**

The expression of ATP23 was significantly lower in tumor tissues, particularly in the CMS4 subtype. No significant correlation was observed between ATP23 expression and clinical characteristics or molecular mutations in COAD. Higher ATP23 levels were associated with improved survival rates in COAD patients. In vitro experiments indicated that ATP23 inhibits the proliferation, migration, and invasion capabilities of COAD cells. Moreover, decreased ATP23 expression may impair oxidative phosphorylation in T cells, contributing to the formation of an immune-evasive microenvironment, and potentially leading to reduced efficacy of both immunotherapy and conventional chemotherapy.

**Conclusions:**

ATP23 is a potential prognostic marker for COAD patients. Reduced ATP23 expression may inhibit oxidative phosphorylation in T cells and contribute to the formation of an immunosuppressive microenvironment.

## Introduction

The current assessment in local recurrence risk and prognosis of colon adenocarcinoma (COAD) primarily relies on tumor-node-metastasis (TNM) staging system (Dekker et al., 2019; Delattre et al., 2022). However, the TNM staging system has certain limitations attributed to the inherent heterogeneity of COAD (Frank et al., 2021). In 2015, a widely acknowledged classification system was introduced to establish the consensus molecular subtypes (CMS) of colorectal cancer (CRC), categorizing patients into four distinct subgroups (CMS1-4) (Guinney et al., 2015). This categorization is primarily based on the RNA expression patterns exhibited by tumors, which can serve as predictive indicators for patient prognosis and the effectiveness of anticancer therapies (Joanito et al., 2022; Martelli, Pastorino & Sobrero, 2022). The CMS4 mesenchymal subtype exhibits several characteristics, including extracellular matrix remodeling, activation of TGF-β signaling pathway, stromal angiogenesis, and displays the highest propensity for metastasis with poor overall survival (OS) (Deng et al., 2021; Guinney et al., 2015). BRAF mutation is an important negative prognostic indicator in CRC (Dekker et al., 2019). The predominant form of this mutation is the BRAF V600E variant, which exhibits an incidence rate ranging from 8% to 13% in CRC cases (Grothey, Fakih & Tabernero, 2021). BRAF mutations are most frequently observed in the CMS1 immune subtype, which is associated with poorer OS and relapse-free survival (RFS), akin to the CMS4 subtype (Angerilli et al., 2022; Ciombor et al., 2022). It remains uncertain whether the OS of CMS4 subtype COAD with BRAF mutations is inferior to that of the other three CMS subtypes also harboring BRAF mutations, and the molecular characteristics underlying this subtype have yet to be elucidated.

The protease ATP23 is a mitochondrial intermembrane space enzyme, initially discovered in Saccharomyces cerevisiae as an essential factor for the processing of subunit 6 within the mitochondrial ATPase complex (Yang et al., 2022). ATP23 serves as a substrate for the mitochondrial disulphide relay system and can undergo complete oxidation even under anaerobic conditions, thereby playing a pivotal role in cellular energy metabolism (Heidorn-Czarna, Maziak & Janska, 2022; Yang et al., 2021). To date, limited research has been conducted on the role of ATP23 in tumor progression.

**Figure 1:**
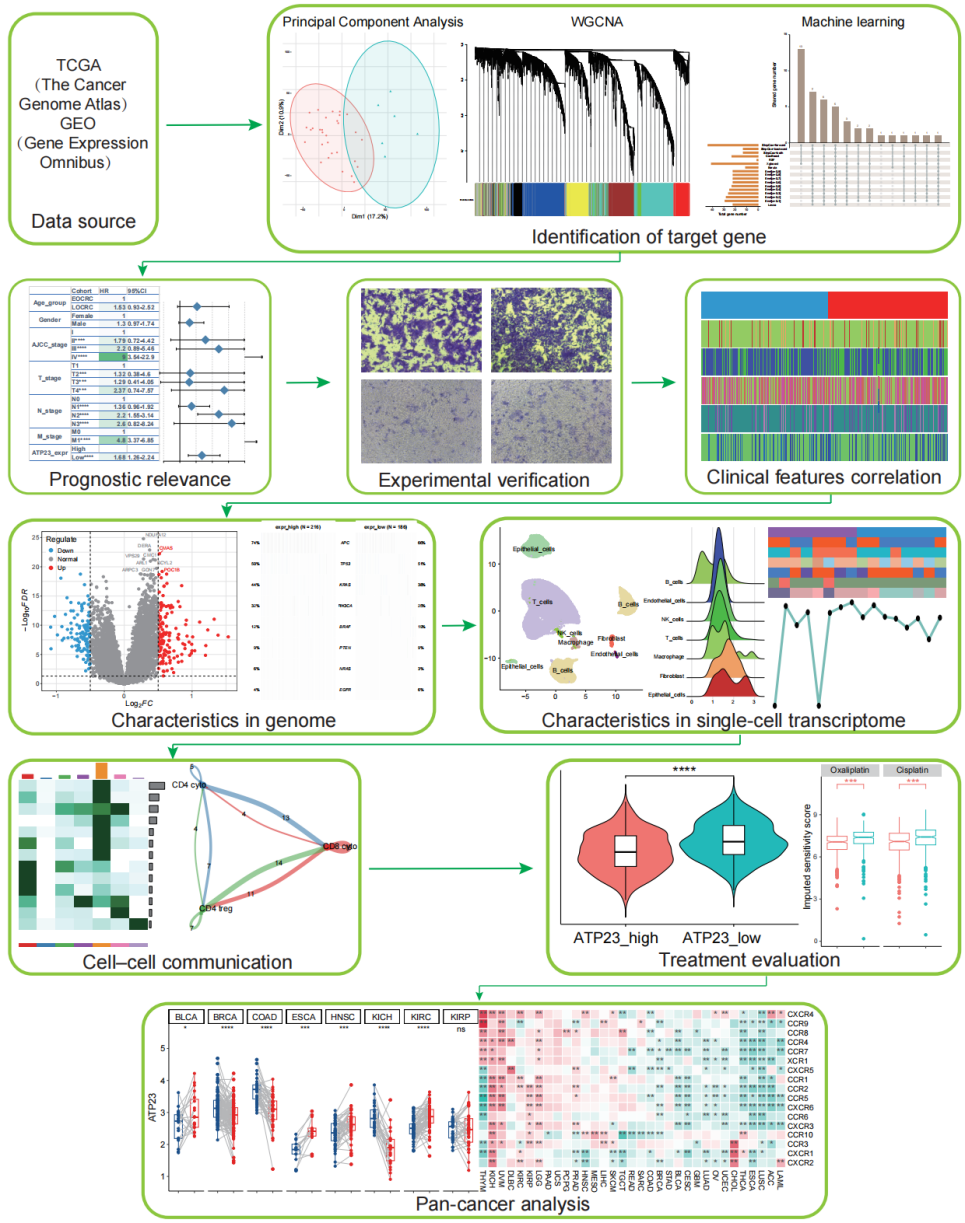
Flowchart of this study.

Using bulk RNA-sequencing and single-cell RNA-sequencing (scRNA-seq) datasets from COAD, this study investigated the expression level and prognostic significance of ATP23 in COAD. In vitro experiments verified the potential roles of ATP23 in COAD development. Additionally, it assessed the correlation between ATP23 expression and clinicopathological features as well as immunotherapy response. Figure 1 presents a workflow diagram illustrating the study process. Our findings suggest that ATP23 can characterize tumor microenvironment (TME) features and predict the antitumor therapeutic efficacy. Decreased levels of ATP23 were associated with unfavorable outcomes, resistance to anticancer therapies, and lack of response to immunotherapy.

## Materials and Methods

### Bulk RNA-sequencing data acquisition and processing

Bulk RNA-sequencing data and corresponding clinicopathological data of patients with COAD were obtained from The Cancer Genome Atlas (TCGA) database (The Cancer Genome Atlas Program (TCGA)-NCI) and the Gene Expression Omnibus (GEO) database (Home-GEO-NCBI (nih.gov/: GSE39582). To validate the robustness of our findings, we utilized three additional datasets from the GEO database, namely GSE44076, GSE14333 and GSE17538. Please refer to Table S1 for detailed information of each dataset. The raw count data from TCGA-COAD was first normalized using the transcripts per million (TPM) method and subsequently underwent log2 transformed, while the data from the GEO database was normalized using log2-normalized counts. The analyses were performed using R (Foundation for Statistical Computing 2020) version 4.4.1, with analysis methods and R packages (*e.g.*, ggplot) implemented accordingly.

### Identification of target gene

The R package “WGCNA” (Langfelder & Horvath, 2008) was utilized to conduct WGCNA analysis on the GSE39582 dataset in order to identify geneset associated with the CMS4 subtype (test group). The molecular subtype classification scheme is detailed in the reference literature associated with the dataset. The initial step involved calculating an appropriate soft threshold β (0.8) to fulfill the criteria for constructing a scale-free network. The weighted adjacency matrix was subsequently converted into a topological overlap matrix (TOM), and dissimilarity (dissTOM) was computed. The module exhibiting the strongest correlation (Correlation coefficient = 0.85) with CMS4 subtype was ultimately determined for further analysis.

The GSE39582 dataset was randomly partitioned into a training set (70%) and an internal validation set (30%) prior to any analysis to prevent information leakage. The genes in the module were sorted in descending order based on their expression levels, and the top 1,000 genes were incorporated into machine learning algorithms. This feature ranking and selection process was strictly confined to the training data to avoid biasing the validation results. We employed ten machine learning algorithms: CoxBoost, elastic net (Enet), generalized boosted regression modeling (GBM), Lasso, partial least squares regression for Cox (plsRcox), Ridge, random survival forest (RSF), StepCox, supervised principal components (SuperPC), and survival support vector machine (survival-SVM). Among these, algorithms such as Lasso and RSF incorporate built-in regularization mechanisms to mitigate overfitting. A total of 101 combinations of these algorithms were arranged in the dataset for variable selection and model construction using the “Mime1” package (Liu et al., 2024). For each model, we calculated its C-index in both the train and validation sets. Subsequently, we ranked the models’ predictive performance based on the average C-index. We selected the intersection gene of all models that exhibited robust performance and clinical translational significance. Finally, based on the median gene expression value, we separated the samples in each set into two groups, and Kaplan–Meier (KM) curve analysis demonstrated a significant impact of gene expression on patient survival.

### Validation of prognostic value

Following verification of the proportional hazards assumption using Schoenfeld residuals, both univariate and multivariate Cox regression analyses were performed to assess the prognostic significance of ATP23 in COAD through the GSE39582 dataset. A comprehensive analysis, including ATP23 expression, age, gender, and tumor stage, was employed to construct the Nomo diagram for assessing prognosis accuracy. The calibration plot curve, time-dependent ROC analyses, and optimism-corrected Harrell’s C-index was utilized to evaluate the accuracy of the constructed Nomo diagram. Subsequently, individual patient risk scores were calculated using the “nomogramFormula” package based on these factors. According to the median risk scores, patients were divided into two groups and subjected to KM survival analysis. Furthermore, the association between ATP23 expression and survival was assessed using the Kaplan–Meier Plotter database (Kaplan–Meier plotter (kmplot.com)). Patients were stratified into high- and low-expression groups based on median ATP23 expression automatically calculated using the web tools. KM survival analysis was then performed for each group.

### Correlation with molecular classification and clinicopathological characteristics

The CIBERSORT algorithm was utilized to calculate the levels of immune cell infiltration based on different ATP23 expressions. Immunoregulatory cells and hallmark gene sets were obtained from CellMarker 2.0 (http://117.50.127.228/CellMarker/). The enrichment scores and correlations were calculated using single-sample gene set enrichment analysis with R package “GSVA”. Subsequently, all associations between gene expression levels and immune cell infiltration scores were re-evaluated through partial correlation analysis, with estimated tumor purity included as a covariate to account for potential confounding effects. Finally, the xCell package was employed to validate the results.

The somatic mutation data of COAD patients were acquired from the TCGA database, and subsequently visualized using the “maftools” package (Mayakonda et al., 2018). All samples were divided into two groups based on the median level of ATP23. Mutation types and frequencies of the most commonly mutated genes in each group were manifested. Additionally, we assessed whether there was a difference in tumor mutation burden (TMB) between the two groups and compared them with data from 33 other cancer types accessible within TCGA database.

To elucidate the biological functions associated with ATP23-related genes, we assessed the clinical and gene expression data derived from the GSE39582 dataset. The “limma” package (v3.64.1) was utilized for analyzing differentially expressed genes (DEGs) based on ATP23 expression levels. DEGs were defined using the criteria of |log2-fold change|≥ 1 and false discovery rate (FDR) ≤ 0.05. The Gene Set Enrichment Analyses (GSEA) were conducted to predict the relevant biological pathways associated with COAD, and the *p*-values are adjusted for multiple comparisons.

Furthermore, using the TIDE algorithm, we computed the dysfunction, exclusion, and TIDE scores for all samples in TCGA-COAD and GSE39582 to evaluate the role of ATP23 in predicting immunotherapy responses. To assess the predictive value of ATP23 expression for chemotherapy drug efficacy in patients with COAD, we utilized the R packages “OncoPredict” (Maeser, Gruener & Huang, 2021) to investigate the correlation between gene expression and IC50 values of commonly used chemotherapeutic agents, including oxaliplatin, cisplatin, 5-fluorouracil, PLX-4720, and dabrafenib (The latter two being selective BRAF V600E inhibitors). The IC50 data was obtained from Genomics of Drug Sensitivity in Cancer (https://www.cancerrxgene.org/). Furthermore, we conducted GSEA to evaluate the association between ATP23 expression levels and pathways related to DNA damage repair (DDR) as well as apoptosis signaling, aimed at identifying the mechanisms underlying its effect on drug activity. The R package “TCGAplot” (Liao & Wang, 2023) was utilized to elucidated the multi-omics landscape of ATP23 in pan-cancer, encompassing the TPM expression matrix, TMB, microsatellite instability (MSI), immune checkpoint genes, and immune score analysis.

### Single-cell RNA-sequencing Data acquisition and processing

Single-cell RNA-sequencing (scRNA-seq) profiles and clinical information of sixteen treatment-naive COAD patient tissue samples and seven adjacent normal colonic tissue samples were obtained from the GSE200997 dataset. We analyzed scRNA-seq data using R package “Seurat” (v4.4.0). The data underwent quality filtering, retaining only cells that expressed ≥ 200 detected genes and genes that were expressed in ≥ 3 cells. Subsequently, in order to eliminate mitochondrial gene interference and doublets, cells were filtered based on the following criteria: percent.mt < 10, nFeature_RNA > 200, and nFeature_RNA < 3000. After performing data standardization, the “Harmony” package was employed to mitigate batch effects. The UMAP dimensionality reduction method was utilized for visualizing the data. Following the clustering process, the scDblFinder package (v1.22) was employed to further eliminate doublets, utilizing the default parameter settings. Cell types were identified based on markers from the CellMarker 2.0 database and relevant literature, while T cell marker genes for subpopulation clustering were obtained from published studies (Zheng et al., 2021). We utilized the “scpubr” package to demonstrate the expression patterns of ATP23 across distinct cell clusters.

### Comprehensive analysis of immune microenvironment characteristics

To assess the relative differences in ATP23 expression between normal and malignant epithelial cells, copy number variation (CNV) analysis was carried out with InferCNV package (v1.24). We used non-malignant cell types (*e.g.*, fibroblasts, endothelial cells, and T cells) from the tumor microenvironment as the reference set, with a cutoff value of 0.1. We performed subpopulation analysis of T cells to assess the impact of high and low ATP23 expression on the distribution of T subpopulations. By confirming the disparities in immune escape molecular profiles between these two subgroups, we further establish the association between ATP23 expression and immune function. To analyze the impact of ATP23 expression on different metabolic pathways, all T cells were stratified into high, medium, and low expression groups according to ATP23 expression levels. Metabolic pathway activity in individual cells was assessed using the AUCell algorithm, based on pathway gene sets derived from KEGG through the scMetabolism package. To further investigate the potential role of ATP23 in T cell differentiation and intercellular communication, we subsequently employed the “CellChat” package (v2.2.0) to elucidate the ligand–receptor interactions and cellular communications within T subpopulations. Finally, we conducted correlation analysis and GSEA to evaluate the association between ATP23 expression levels and markers of T cell activity, as well as relevant functional gene sets.

### Cell lines, reagents, and antibodies

HCT116 and HT29 were purchased from the FuHeng Inc. (Shanghai, China) and cultured in McCoy’s 5a (KeyGEN, China) supplemented with 10% fetal bovine serum (FBS, ExCell, China) at 37 °C with 5% CO_2_. Anti-ATP23 (A12885) and anti-β-actin (AC004) antibodies were obtained from ABclonal (Wuhan, China). Anti-Rabbit secondary (RGAR001) and anti-Mouse secondary (RGAM001) antibodies were obtained from Proteintech.

### RNA interference and lentivirus infection

Specific ATP23 small interfering RNA (siRNA) was purchased from Gene&Bio Co. Ltd (Shandong, China). Cells were transfected with 50 nmol/L siRNA #1, siRNA #2 or negative control (NC) siRNA using the OriGene transfection reagentt.

### Cell proliferation assays

The Cell Counting Kit -8 (CCK-8; GlpBio, Montclair, CA, USA) was employed to assess cell viability. Cells were seeded in 96-well plates at a density of 2,000 cells per well. After incubation for the designated time points (0, 1, 2, 3, or 4 d), 10 μL of sterile CCK-8 was added to each well and incubated for another 2 h at 37 °C. Absorbance was then determined at 450 nm (Tecan, Switzerland).

### Cell migration

To assess cell migration, we performed a wound-healing assay. We seeded 1 × 10^6^ cells per well in 6-well plates for 24 h. After drawing a reference line on the plate bottom, we created three parallel scratch wounds perpendicular to the line with a sterile pipette tip. We then gently washed the wells twice with PBS and added three mL of fresh medium. Using an inverted microscope, we imaged the wounds at 0 h, 24 h and 48 h. The scratch area was measured by ImageJ software. Transwell assays were conducted using a 24-well plate system with transwell chambers (Corning, NY, USA). A total of 2 × 10^5^ transfected cells were suspended in 100 μL of culture medium and seeded into the upper chamber, while 600 μL of culture medium supplemented with 10% FBS was added to the lower chamber. After incubation at 37 °C, 5% CO_2_ for 48 h, the cells in upper chamber were washed and the chamber was fixed in 4% paraformaldehyde for 30 mins, then stained with 0.1% crystal violet for 30 s. Three randomly selected views were counted for statistical analysis per well.

### Clonogenic assays

We evaluated the clonogenic potential of HCT116 and HT29 cells by seeding 900 cells per well in 6-well plates. We refreshed the culture medium every two days during the 10-14 days incubation period. After the culture, we fixed the colonies with 4% paraformaldehyde and stained them with 0.1% crystal violet, each step lasting for 30 mins at room temperature. The colony formation rate was calculated as (Number of colonies/900) × 100%.

### Western blot

Proteins were extracted from the HCT116 and HT29 cells, and the concentrations were measured using a BCA protein assay (Solarbio, Beijing, China). Proteins were separated using sodium dodecyl sulfate–polyacrylamide gel electrophoresis and transferred to a polyvinylidene fluoride membrane (Millipore, USA). The membranes were incubated with the primary antibodies for 16 h (4 °C) and the secondary antibodies for 1 h (room temperature) and subsequently washed three times with tris-buffered saline plus Tween^®^20. The membrane was imaged with ECL reagents (Cell Signaling Technology, Danvers, MA, USA) by the imaging system (Amersham, Chicago, IL, USA). Figure S1 shows representative western blots of three independent experiments for two cell lines.

### Quantitative real-time polymerase chain reaction

We extracted total RNA from HCT116 and HT29 cells with a commercial kit (Solarbio, Beijing, China) and measured its concentration using a NanoDrop One (Thermo Fisher Scientific, USA). Using Super M-MLV reverse transcriptase (TransGen Biotech, Beijing, China), we reverse-transcribed the RNA into cDNA. We then performed qPCR on a Roche fluorescence quantitative system with a SYBR Green kit (TransGen Biotech), according to the manufacturer’s instructions. We analyzed the data *via* the 2−ΔΔCT method to determine relative mRNA expression, with all primers listed in Table 1.

**Table 1:** Primer sequences utilized for qRT-PCR analysis.

Primer	Sequence
ATP23	F: ATGACCTGGAGGCTGTGGTTCCACA
	R: CAAGCAGAAGTTTGACATA
E-cadherin	F: AAAGCCTCAGGTCATAAACATC
	R: CGCCTCCTTCTTCATCATAG
N-cadherin	F: CAGGTTTGGAATGGGACAGT
	R: GTAGGATCTCCGCCACTGAT
vimentin	F: ACCGCTTCGCCAACTACATC
	R: TTTCCTCTTCGTGGAGTTTCTT
β-actin	F: GGGAAATCGTGCGTGACATT
	R: GGAAGGAAGGCTGGAAGAGTG

### Cell inhibition assays

Cells were seeded into 96-well plates (6,000∼10,000 cells/well), and treated with cisplatin at different concentrations (20, 30, 40, 50, 60, 80, and 100 μM) for 24 h. The growth inhibition rate for each was calculated as (1-OD value of the treatment group/OD value of the control group) ×100%, and IC50 values were determined using GraphPad Prism.

### Assessment of mitochondrial function

The JC-1 assay kit (Cat# C2006; Beyotime, Shanghai, China) was used to assess mitochondrial membrane potential. A one mL working solution of JC-1 was prepared and thoroughly mixed. Cells were incubated with the staining solution for 20 mins at 37 °C in a cell incubator, followed by removal of the supernatant and two washes with 1 × JC-1 staining buffer. Fresh culture medium was then added, and mitochondrial function was evaluated using a fluorescence microscope (Thermo Fisher Scientific; Waltham, MA, USA) at ×400 magnification. The mean fluorescence intensity was calculated according to the fluorescence ratio (590 nm/530 nm) for JC-1. To further evaluate mitochondrial function, MitoSOX Red probe (Cat# S0061S; Beyotime, Shanghai, China) was employed to detect mitochondrial superoxide levels. The MitoSOX Red working solution was added and cells were incubated at 37 °C for 30 mins, after which nuclei were counterstained with Hoechst. Subsequently, the Mito-Tracker Red CMXRos working solution was applied, replaced with culture medium, and cells were observed under a fluorescence microscope at ×400 magnification. The level of mitochondrial superoxide was determined based on the intensity of red fluorescence. All the images were analyzed using ImageJ software. Each experimental condition was performed with *n* = 3 biological replicates. Additionally, ATP levels in cell homogenates were quantified using an ATP assay kit (Cat# S0026; Beyotime, Shanhai, China) according to the manufacturer’s instructions. The relative ATP level was calculated according to the following formula: relative ATP level = ATP value/protein value. Each experimental condition was replicated *n* = 3 times biologically.

### Statistical analysis

The relative differences in ATP23 expression between different groups were determined using the Welch’s *t*-test for two groups and one-way ANOVA analysis for multiple groups. Survival analyses, including OS, post-progression survival (PPS), and relapse-free survival (RFS), were performed using the Kaplan–Meier method. The association between ATP23 expression and immune cell infiltration in COAD was assessed using Spearman’s correlation analysis. Graphical representations and statistical computations were conducted using the R software package. Experiment data were shown as the mean ± SD. The significance of the differences between groups was verified using ordinary one-way analysis of variance. Statistical analyses were conducted using SPSS 22.0 (IBM, Armonk, NY, USA). Additionally, we obtained a copyright license for GraphPad Prism (version 8.01; GraphPad software) and used it for graphic work and statistical analysis. FDR correction was employed to adjust *p*-values across all multiple-hypothesis testing procedure. This encompassed analyses of module–trait correlations, DEGs, TIDE, univariate and multivariate Cox models, copy number variation, pathway enrichment, chemotherapy drug efficacy, and cellular communications. The statistical significance of differences was determined at a *p*-value threshold of 0.05.

**Figure 2:**
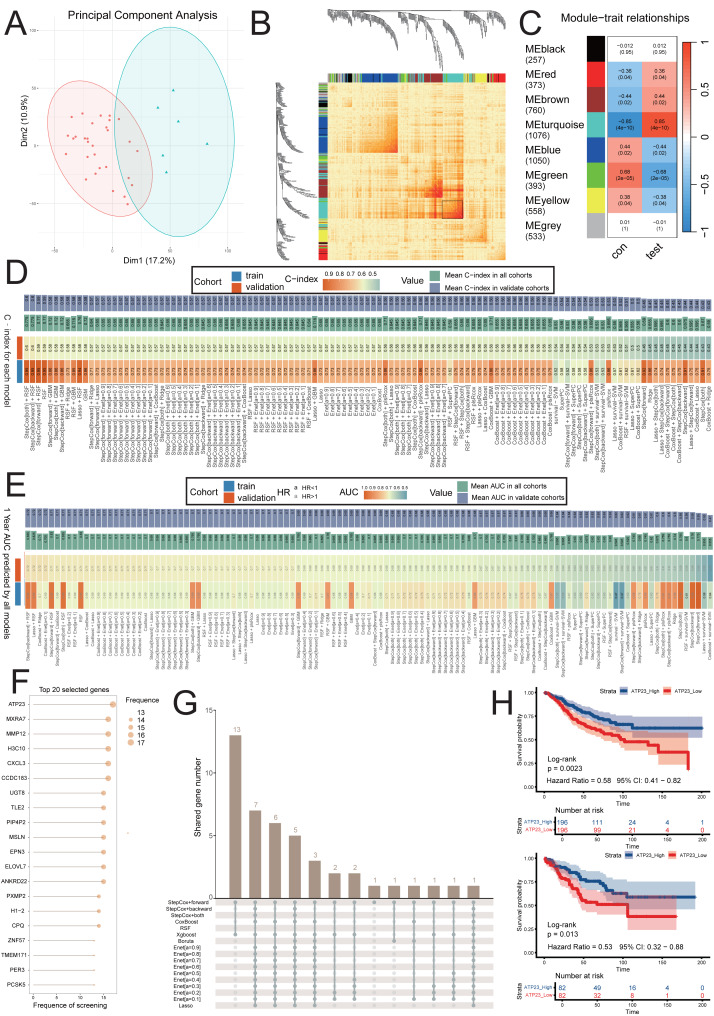
ATP23 was associated with CMS4 subtype in patients with colon adenocarcinoma (COAD) harboring BRAF mutations. (A) The samples in experimental group (test group, blue triangle) and control group (con group, red circle) were determined using principal component analysis and clusteranalysis. (B) The optimal geneset was determined through weighted gene coexpression network analysis, with a black box marking this geneset. (C) According to the heatmap of module-trait relationships, the MEturquoise module (comprising 1,076 genes) demonstrated the highest correlations with CMS4 subtype. In each colored module, the number above is the correlation coefficient, and the number in parentheses below is the FDR -adjusted *P*-value. (D) Analysis of prognosis-related genes using a combination of 101 machine learning algorithms. The optimal prognostic geneset comprising 20 genes was determined using the StepCox[both] + RSF model. (E) The heatmap illustrated the AUC values for predicting 1-year survival in both the train and test datasets across all models. AUC, area under curve. (F) The candidate genes were identified through the intersection of genesets determined by predictive models. (G) The contribution of the 20 genes identified by different models. (H) The Kaplan Meier plot showing the overall survival of patients in both datasets stratified by ATP23 expression.

## Results

### ATP23 is closely associated with poor prognosis in patents with CMS4 subtype colon adenocarcinoma

To identify modules significantly associated with CMS4 subtype of COAD with BRAF mutations, we performed WGCNA analysis on the GSE39582 dataset. The construction of a co-expression network was carried out using 36 COAD samples with BRAF mutations, excluding any outlier samples (Fig. 2A). We used the scale-free topology fit plot and mean connectivity plot to demonstrate the appropriateness of the chosen power (Figs. S2A–S2B). By setting the minimum module gene count to 50 and mergeCutHeight to 0.25, a total of eight modules were obtained (Fig. 2B). Our findings revealed a strong correlation between the MEturquoise module and the CMS4 subtype (cor = 0.85, Figs. 2C and S2C). The positive correlation (*r* = 0.482) in the GS-MM plot indicated that trait-associated genes serve as central hubs within the module (Fig. S2D). Detailed relative expression values of each gene in the turquoise module are illustrated in Table S2. We arranged the 1,076 genes based on their expression levels, ranked them in descending order, and selected the top 1,000 genes. Subsequently, we employed a combination of 101 machine-learning algorithms to analyze the prognostic potential of these 1,000 genes using the GSE39582 dataset. In the train set, we fitted each prediction model with a tenfold cross-validation framework and computed the C index and 1-year area under the curve (AUC) value in both the train and validation sets, as illustrated in Figs. 2D–2E. Through comprehensive screening, we identified StepCox[both] + RSF as the predictive model with exceptional accuracy and translational relevance, incorporating a total of 20 genes (Fig. 2F). Through sorting occurrence frequency of these 20 genes in all models (Figs. 2F–2G), ATP23 was selected as the target gene associated with CMS4 subtype and poor prognosis in COAD patients with BRAF mutations. Figure 2H indicates that low expression of ATP23 is correlated with unfavorable prognosis for COAD patients in both the train and validation sets, as demonstrated by KM-plot analysis.

**Figure 3:**
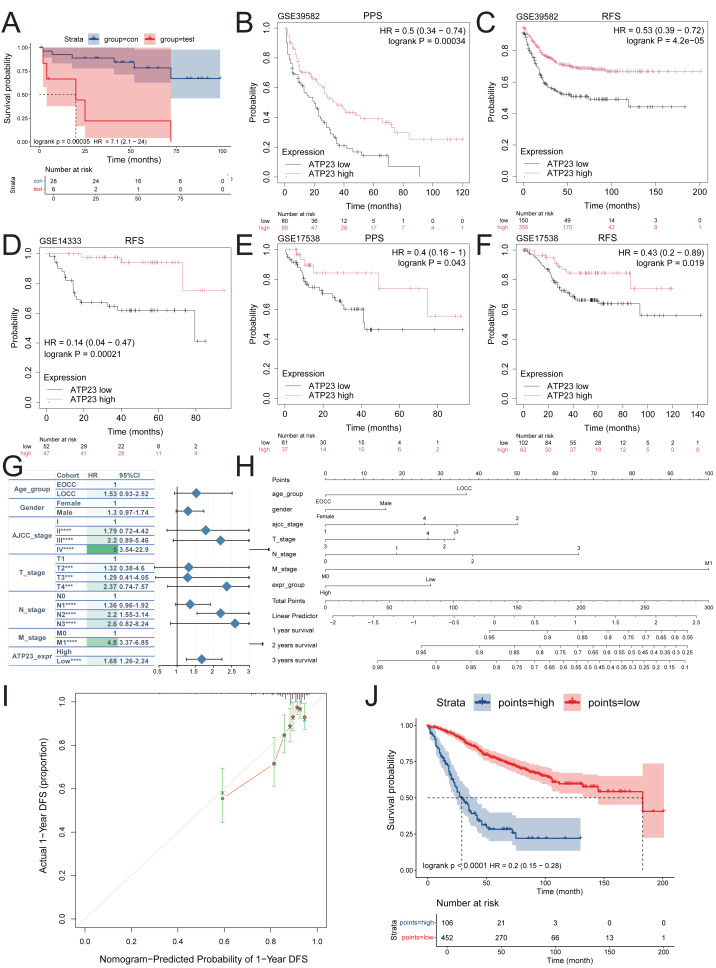
Low expression of ATP23 was associated with poor prognosis of COAD patient. (A) The overall survival (OS) of CMS4 subtype patients in COAD was worse than that of patients from other three molecular subtypes. (B–F) COAD patients with low ATP23 level exhibited significantly shorter post-progression survival (PPS) and recurrence-free survival (RFS) compared to those with high ATP23 expression. (B) PPS in GSE39582; (C) RFS in GSE39582; (D) RFS in GSE14333; (E) PPS in GSE17538; (F) RFS in GSE17538. (G) Univariate COX regression analysis showing ATP23 as independent prognostic factors of COAD in GSE39582. AJCC, American Joint Committee on Cancer; EOCC, early-onset colon cancer; LOCC, late-onset colon cancer. (H) The clinicopathological features and ATP23 expression were assessed using a nomogram to accurately predict the prognosis of patients with COAD. (I) The calibration curves for the nomogram. DFS, disease-free survival. (J) Kaplan–Meier survival plots of risk stratification according to the nomogram total points. (*** *p* < 0.005, **** *p* < 0.001).

### ATP23 may serve as an independent poor prognostic factor for COAD patients

The results depicted in Fig. 3A confirm that the OS of CMS4 subtype COAD with BRAF mutations is significantly inferior compared to the other three CMS subtypes, which also harbor BRAF mutations. As previously mentioned, this poor prognosis may be attributed to aberrant ATP23 expression. To validate this point, the Kaplan–Meier Plotter database was used to examine the correlation between ATP23 and survival prognosis of COAD patients, inclusive of those with and without BRAF mutations and encompassing all molecular subtypes. Low ATP23 expression was found to be a significant predictor of poor PPS in the GSE39582 and GSE17538 datasets, as well as for RFS in the GSE39582, GSE14333, and GSE17538 datasets (Figs. 3B–3F). Furthermore, ATP23 was included in the Cox regression analysis. Based on the results of the proportional hazards residual analysis (Fig. S3A), a univariate analysis was conducted by integrating age, gender, TNM stage, and ATP23 expression levels (Fig. 3G). Multivariable regression analysis results are summarized in Fig. S3B. The findings indicated that ATP23 may serve as a valuable prognostic biomarker. The model’s predictive accuracy, as measured by the C-index, was 0.68 after correction for optimism. Subsequently, these risk factors were integrated into a nomogram model for the prediction of 1-, 3-, and 5-year overall survival rates (Fig. 3H). The calibration curves demonstrated favorable discrimination and calibration of the nomogram model (Figs. 3I and S3C). In addition, time-dependent ROC analyses at 36, 60, and 90 months are provided in Fig. S3D. According to the nomogram scoring system, each patient was assigned a total score and then classified into either the high-nomogram or low-nomogram group based on the median score. Patients in the high-nomogram group demonstrated an unfavorable prognosis (Fig. 3J).

### ATP23 inhibited the progression of COAD *in vitro*

**Figure 4:**
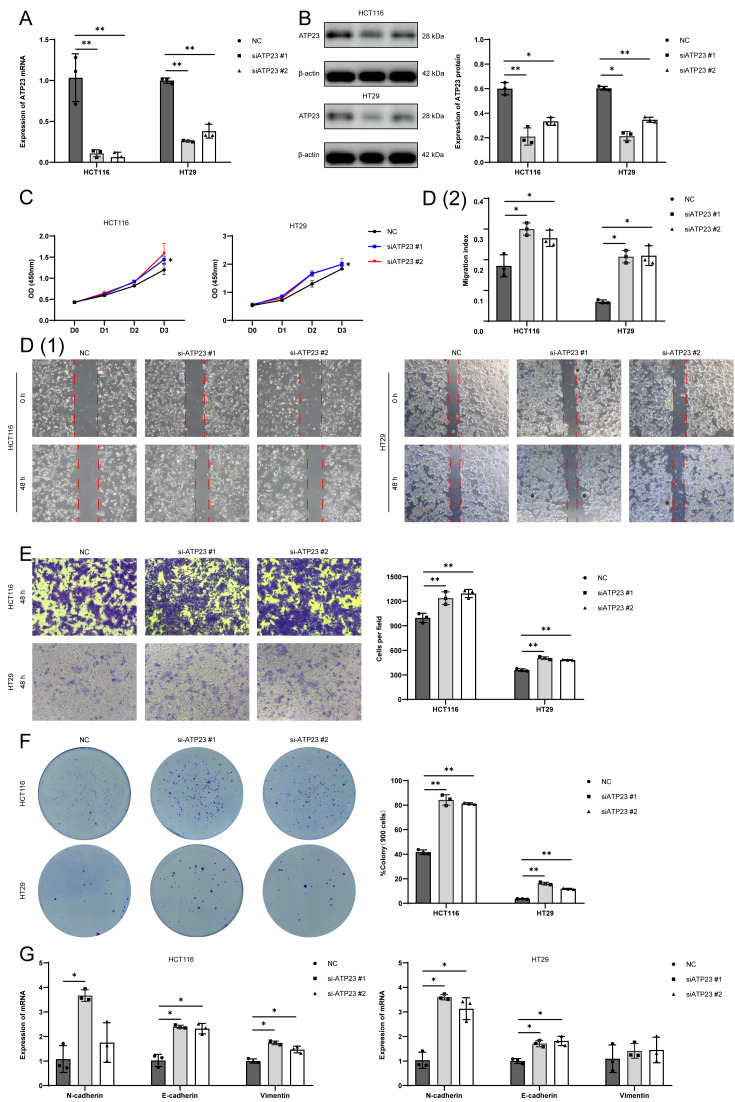
ATP23 inhibits proliferation, migration and invasion in both HCT116 and HT29 cell lines. (A) The qRT-PCR analysis of the expression of ATP23 in HCT116 and HT29 cell lines transfected with NC or siATP23. (B) Western blot analysis of ATP23 protein expression, with quantification performed using grey analysis. (C) Proliferation rates assessed by CCK-8 assays. (D) Scratch wound healing assays to evaluate migratory ability, with closure areas quantified relative to the original wound area using ImageJ software. Scale bar: 200 µm. (E) Transwell assay was applied to evaluate cell migration and invasion ability. Scale bar: 80 µm. (F) Colony formation assays to assess proliferation capacity. (G) The mRNA expression levels of E-cadherin, N-cadherin, and vimentin were determined by qRT-PCR (* *p* < 0.05, ** *p* < 0.01, compared with the NC group; *n* = 3).

To further investigate the roles of ATP23 in COAD development, we constructed ATP23 knockdown cell lines by transfecting siRNA duplexes. In both HCT116 and HT29 cell lines, ATP23 mRNA levels was efficiently knocked down by two distinct siRNAs (Fig. 4A). Western blot analysis confirmed these findings, showing a consistent reduction in ATP23 protein expression (Fig. 4B). CCK-8 assays revealed that ATP23 knockdown significantly enhanced the proliferative capacity in both cell lines (Fig. 4C). To assess the impact of ATP23 on cell migration, wound healing (Fig. 4D) and transwell (Fig. 4E) assays were conducted. Both experiments demonstrated that ATP23 suppression markedly promoted cancer cell migration. Clone formation assays in both cell lines indicated that ATP23 knockdown increased the proliferative potential of cancer cells (Fig. 4F). The results of qRT-PCR (Fig. 4G) showed that ATP23 knockdown decreased the expression of E-cadherin while increasing N-cadherin and vimentin levels, suggesting an enhancement in invasion ability. Alternatively, we employed a plasmid-mediated approach to overexpress ATP23 in HCT116 and HT29 cell lines (Fig. S4A). Functional assays, including the CCK-8 (Fig. S4B), wound healing (Fig. S4C), and colony formation assays (Fig. S4D), demonstrated that ATP23 upregulation significantly suppressed proliferation, migration, and clonogenic capacity in COAD cells. Additionally, qRT-PCR analysis indicated that ATP23 overexpression enhanced E-cadherin expression while reducing the levels of N-cadherin and vimentin (Fig. S4E). Collectively, these findings indicate that ATP23 may inhibit the proliferation, migration, and invasion capabilities of COAD cells.

### ATP23 expression is downregulated in tumor tissue and positively correlated with the majority of T cell subpopulations

**Figure 5:**
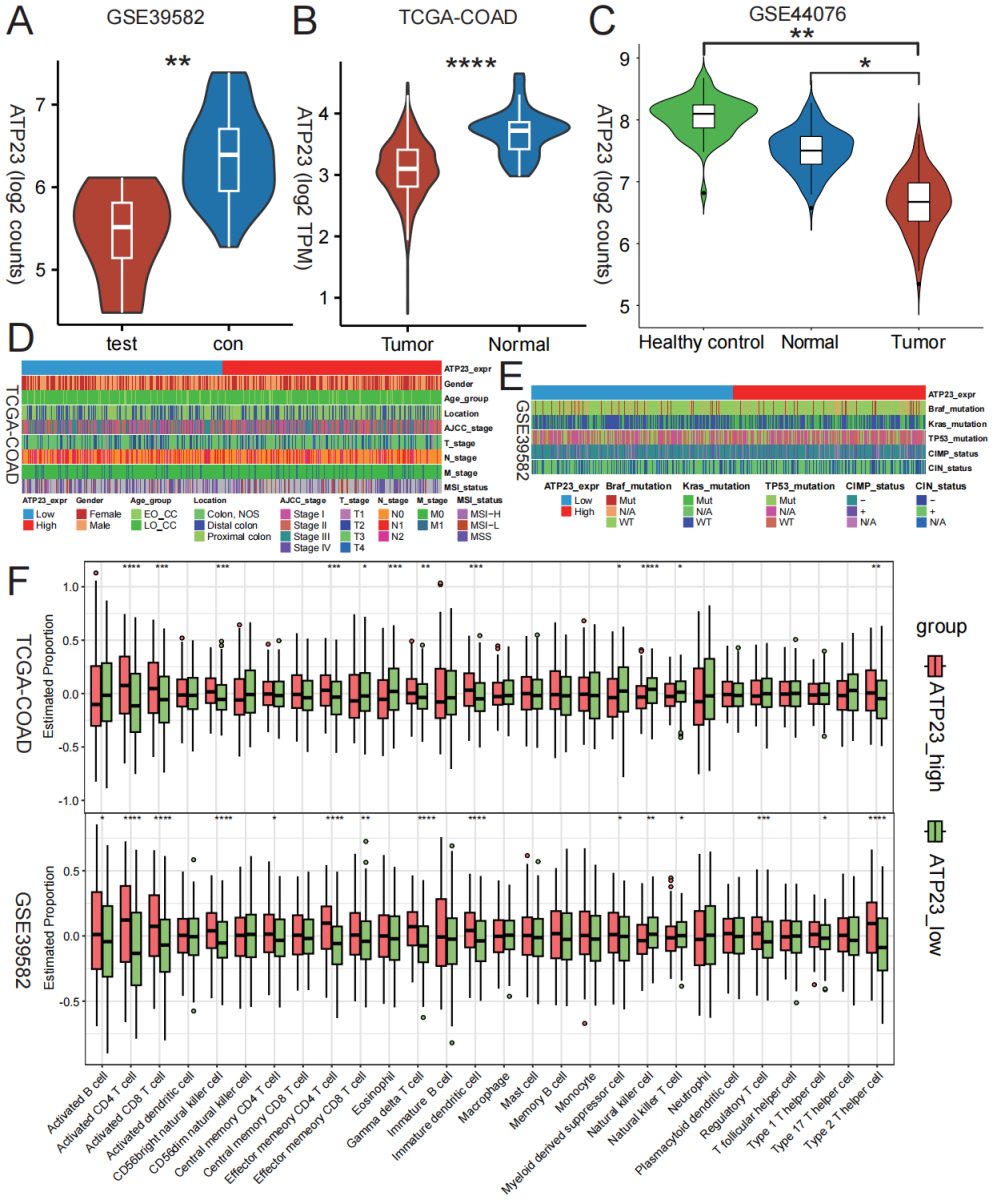
ATP23 manifested a subdued expression profile within tumor tissues and displayed a positive association with the infiltration magnitudes of diverse T cell subpopulations in COAD. (A) The expression level of ATP23 was significantly downregulated in the CMS4 subtype COAD tissues harboring BRAF mutations in GSE39582 (test, *n* = 6; con, *n* = 30). (B) ATP23 expression was lower in tumor tissues compared to adjacent normal tissues in TCGA-COAD dataset (Tumor, *n* = 473; Normal, *n* = 41). (C) Compared to paired adjacent normal tissues and healthy control tissues, the expression of ATP23 was dramatically lower in tumor tissues (Healthy control, *n* = 50; Normal = 98; Tumor, *n* = 98). (D–E) No correlation found between ATP23 expression, clinical characteristics, and molecular mutation signatures in COAD. (D) TCGA-COAD; (E) GSE39582. (F) Correlation analysis between immune cell infiltration, adjusted by tumor purity, and ATP23 expression level. TCGA-COAD (ATP23_high, *n* = 235; ATP23_low, *n* = 238); GSE39582 (ATP23_high, *n* = 283; ATP23_low, *n* = 283) (* *p* < 0.05, ** *p* < 0.01, *** *p* < 0.005, **** *p* < 0.001).

As reduced ATP23 expression has been linked to a poor prognosis of CMS4 subtype, we conducted further analysis of its expression levels and correlation with phenotype in COAD tissues. The expression of ATP23 was significantly downregulated in CMS4 subtype compared to the CMS1-3 subtypes (Fig. 5A). Moreover, its expression level in tumor tissue was significantly lower than that in adjacent normal tissue and healthy control colon tissue (Figs. 5B–5C). Interestingly, there was no significant correlation between ATP23 expression and gender, age, tumor location, TNM stage, molecular characteristics or genomic instability of the tumor (Figs. 5D–5E). Therefore, we endeavored to elucidate the underlying factors by which ATP23 affects patient prognosis. Previous research has shown that tumor-associated immune cells are a crucial component of TME, which is strongly associated with patient prognosis (Kim et al., 2019). We observed a positive correlation between ATP23 expression and increased infiltration levels of the majority of T cell subpopulations within the colon cancer microenvironment (Fig. 5F and Figs. S5A–S5C).

### ATP23 affects cellular oxidative phosphorylation

**Figure 6:**
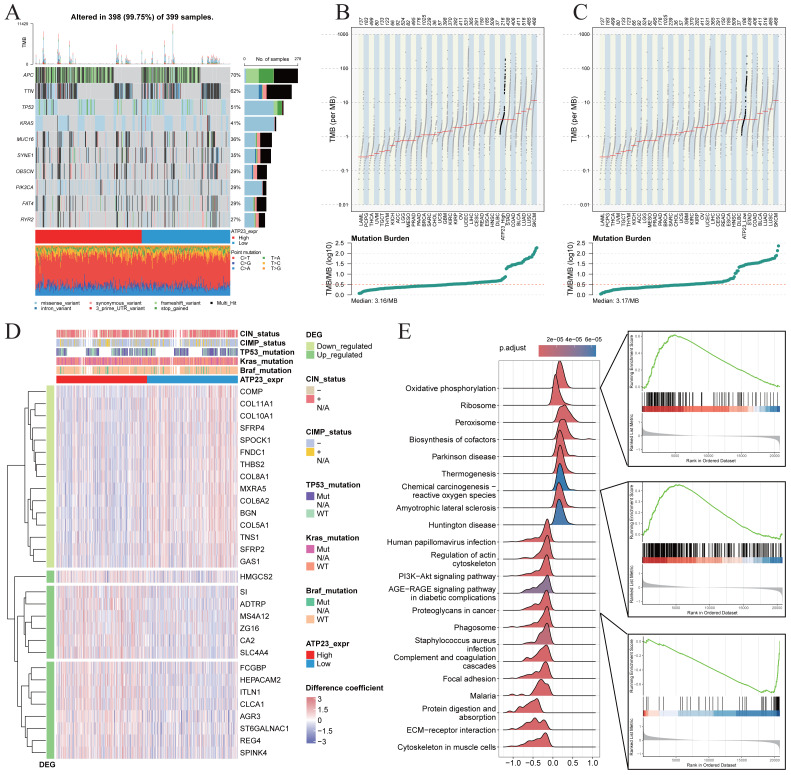
ATP23 expression showed no association with tumor mutation burden (TMB), but exhibited a positive correlation with levels of oxidative phosphorylation. (A) The comparison of mutation signature and TMB in TCGA-COAD dataset between patients with high and low ATP23 (ATP23_expr High, *n* = 200; ATP23_expr Low, *n* = 199). (B–C) The differences in the TMB value between COAD patients with high and low ATP23 and compared to other 33 cancer types in TCGA database. (B) ATP23 high group; (C) ATP23 low group. (D) Heatmap showing analysis of differential expression of top 30 ATP23-related genes in GSE39582. (E) The GSEA analysis revealed a positive correlation between ATP23 expression, oxidative phosphorylation levels, and chemical carcinogenesis-reactive oxygen species, while indicating a negative association between ATP23 expression and cancer proteoglycans.

The previous mention of the absence of any correlation between ATP23 expression and the molecular characteristics of the tumors has prompted us to further investigate the disparities in genomic mutations among ATP23 subgroups. Consequently, we delineated a comprehensive mutation landscape that distinguishes high-ATP23 from low-ATP23 groups and observed no discernible variation profile or TMB between these two groups (Fig. 6A). The median TMB values were also computed for both groups and compared with those of 33 tumors in the TCGA database. Once again, no statistically significant differences were observed between these two groups (Figs. 6B–6C).

The top 30 DEGs between the high- and low-ATP23 groups in the GSE39582 dataset were illustrated in Fig. 6D (For more detailed information on DEGs, please refer to Table S3). It was observed that the low-ATP23 group exhibited enrichment of genes belonging to the Collagen family, which play crucial roles in tumor microenvironment modulation, extracellular matrix (ECM) remodeling, immune suppression, and metastasis (Necula et al., 2022; Sorushanova et al., 2019). Meanwhile, the high-ATP23 group exhibited enrichment of multiple genes, such as HMGCS2, SI, MS4A12 and ZG16 *etc*., which are well-known for their involvement in the regulation of metabolism, cellular energy homeostasis, and immunity (Asif et al., 2022; Danialifar et al., 2024; Meng et al., 2021; Suenaga et al., 2021). Further GSEA analysis revealed a positive association between ATP23-related genes and oxidative phosphorylation as well as chemical carcinogenesis-reactive oxygen species (ROS), while showing a negative correlation with proteoglycans in cancer as well as ECM-receptor interaction (Fig. 6E). The detailed GSEA results are shown in Table S4. To summarize, ATP23 and its associated genes may enhance the infiltration of tumor-related immune cells by regulating intracellular oxidative phosphorylation and ECM remodeling, thereby significantly impacting patient prognosis and survival. Next, we would validate this hypothesis by scRNA-seq data in COAD samples.

### ATP23 expression profiles among different cell types

**Figure 7:**
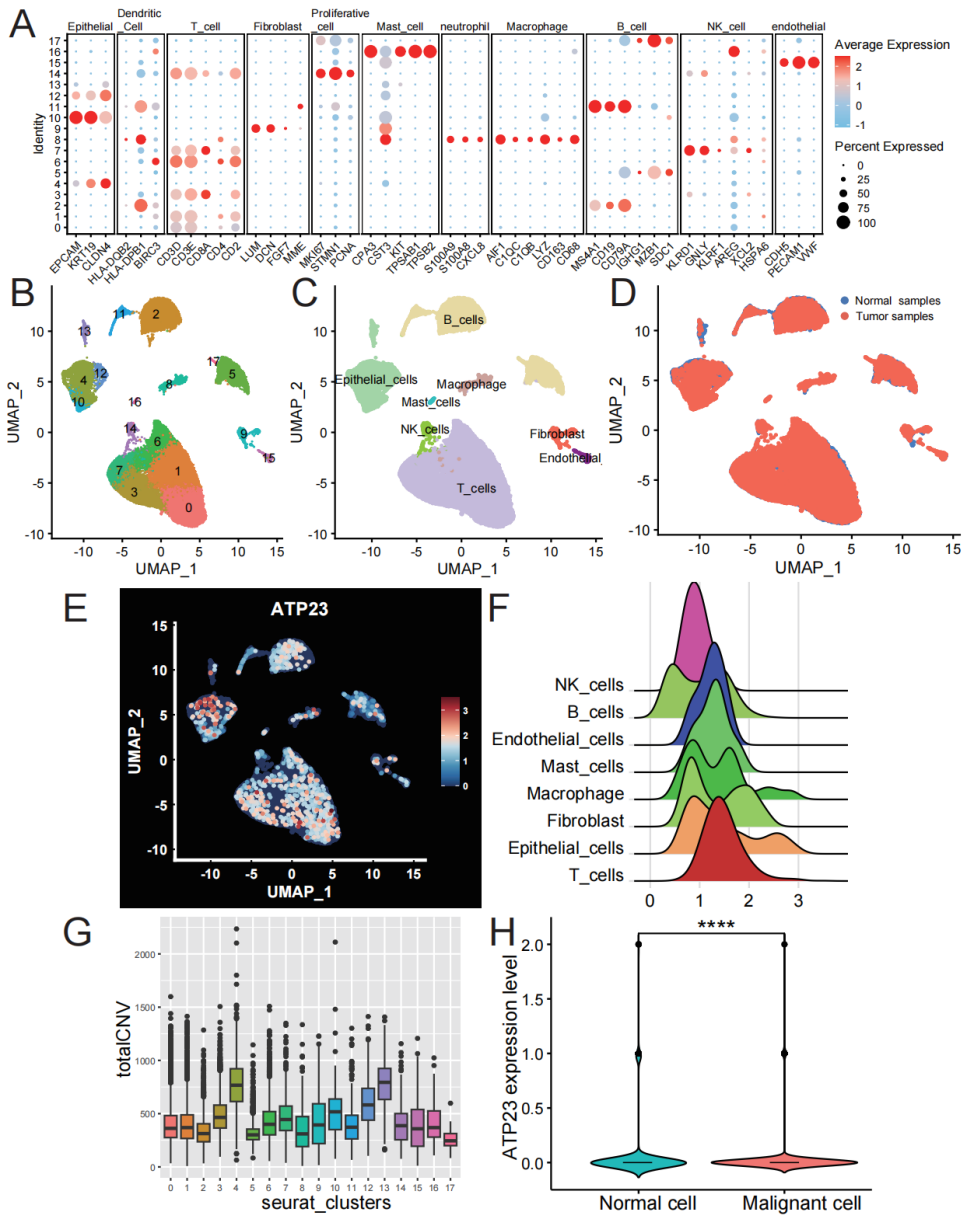
ATP23 was diffusely expressed across various cell types, with higher levels detected on benign colon epithelial cells compared to malignant epithelial cells. (A) The cell identities were annotated based on marker genes derived from CellMarker 2.0 database and relevant literature. (B) Uniform manifold approximation and projection (UMAP) plot of 37, 388 cells colored by seurat clusters. (C) UMAP diagram showing eight subsets labeled with cell type annotation. (D) UMAP plot displayed the distribution of tumor samples and normal samples. (E–F) The expression levels of ATP23 in different cell types. (E) UMAP plot; (F) Ridge plot. (G) Comparative analysis of CNV levels varying degrees of malignancy among different seurat clusters. (H) Malignant epithelial cells exhibited low expression levels of ATP23 in COAD (Malignant cells, *n* = 2, 014; Normal cells, *n* = 2, 489) (** *p* < 0.01; **** *p* < 0.001).

To investigate ATP23 expression in different cell types, we acquired data from the scRNA-seq dataset GSE200997. After applying quality control (Fig. S6A), dimensionality reduction and batch effect elimination, a total of 20, 971 RNA transcripts were identified from 37, 388 cells. Cell types were identified through UMAP clustering followed by manual annotation using established marker genes (Fig. 7A). A total of eighteen cell clusters (Fig. 7B) were categorized into eight distinct cell types: B cell, endothelial cell, epithelial cell, fibroblast, macrophage, mast cell, NK cells, and T cell (Fig. 7C). The majority of the cells (61.61%) originated from tumor samples (Fig. 7D). As depicted in Figs. 7E–7F, ATP23 is diffusely expressed across various cell types. However, its expression level is relatively low in certain epithelial cells (cluster 10). To clarify the disparities in ATP23 expression between normal and malignant epithelial cells, we assessed chromosomal CNVs using transcriptome data (Fig. 7G). Notably, malignant epithelial cells exhibited significantly diminished levels of ATP23 expression compared to their normal counterparts (Fig. 7H).

### Low ATP23 expression characterizes tumor immune escape phenotype

**Figure 8:**
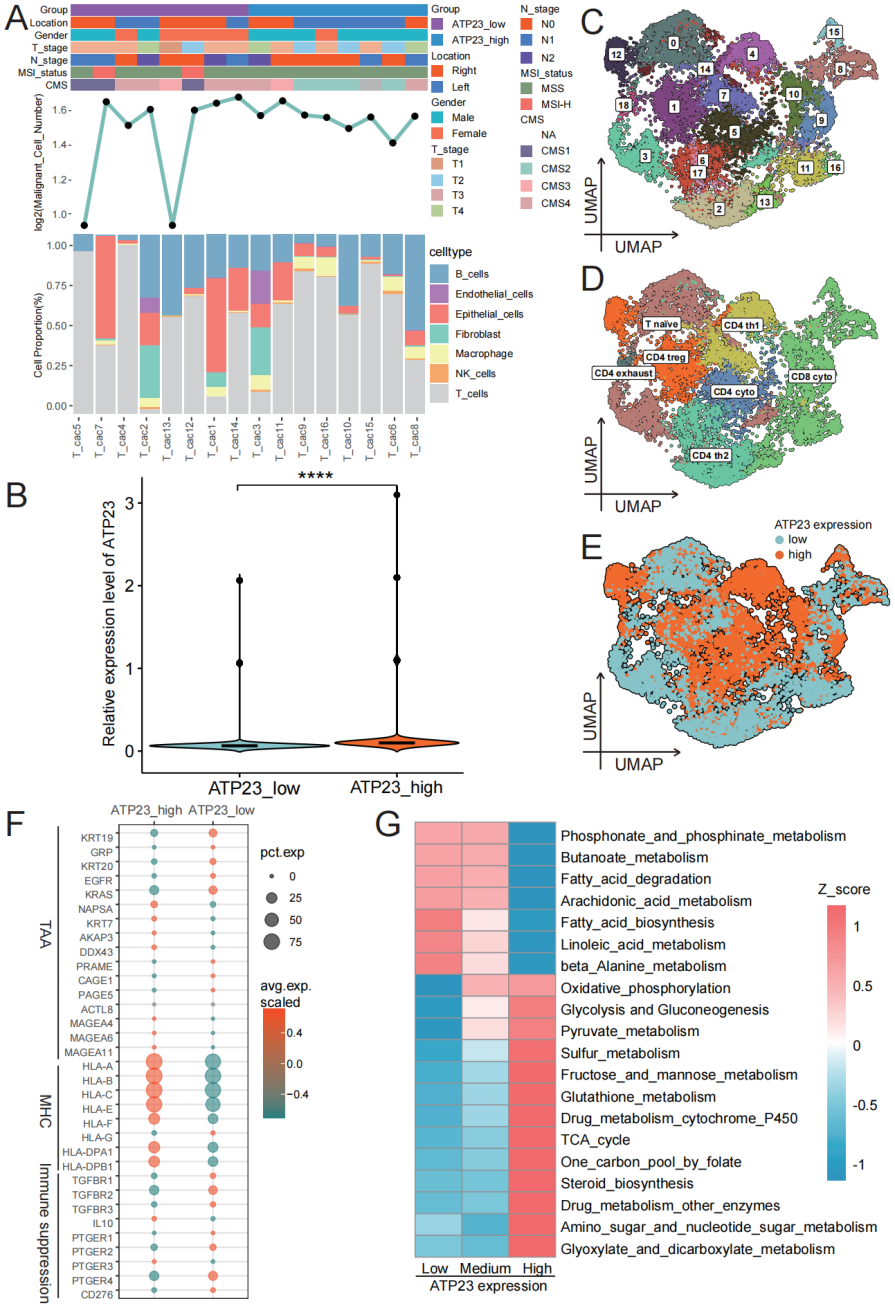
Downregulated ATP23 was associated with the immunosuppressive microenvironment in COAD. (A) Clinical and molecular characteristics of the 16 COAD samples (GSE200997), and the quantity of malignant cells and the distribution of different cell types in each sample. (B) Verification of gene expression levels in ATP23 high and low expression cohorts (ATP23_low, *n* = 12, 885 cells; ATP23_ high, *n* = 10,148 cells). (C) UMAP plot of 13, 199 T cells colored by Seurat clusters. (D) The distribution of different T cell subpopulations annotated with cell type labeling. (E) UMAP plot illustrated the distribution of low and high ATP23 expression in T cell subpopulations. (F) Changes in immune escape molecular profiles of 16 tumor samples between subgroups with high and low ATP23 expression (ATP23_low, *n* = 7,088 cells; ATP23_ high, *n* = 6,111 cells). (G) Heatmap illustrating the impact of ATP23 expression on T cell metabolic pathway activity levels (**** *p* < 0.001).

To investigate the correlation between ATP23 and the TME, we initially isolated 16 tumor samples and analyzed them individually (See Table S5 for details.). As depicted in Fig. 8A, eight of the samples (50.0%) were located in the right colon. Six samples exhibited CMS4 characteristics, while nine belong to CMS1-3 subtypes, and one sample could not be classified. Except for a remarkably low count in two samples, there were no significant differences observed in the number of malignant cells between the samples. In contrast, noticeable variations were evident in the proportions of cell types across different samples, especially T cells. Thus, the heterogeneity of cell types was the main contributor to the interpatient heterogeneity. According to the transcriptome results, we subsequently categorized all samples into two groups based on the average ATP23 expression level: the ATP23_low group and the ATP23_high group. Figure 8B confirmed the difference in ATP23 levels between the two groups. Then, we performed a T cell subpopulation analysis according to marker genes. A total of nineteen cell clusters (Fig. 8C) were categorized into seven distinct T cell subpopulations (Fig. 8D). Each subpopulation exhibited varying levels of ATP23 expression, with CD4 th1 displaying the highest expression level, followed by CD4 cyto, CD4 treg, and CD8 cyto (Fig. 8E).

Given that immune escape is a critical determinant of the immune system’s inability to control tumor progression (Shimizu et al., 2018), we investigated whether ATP23 could modulate immune escape based on scRNA-seq data. Figure 8F demonstrates alterations in the molecular profiles of immune escape between the high and low ATP23 groups. The downregulation of major histocompatibility complex (MHC) molecules in T cells was observed in the ATP23_low group. These findings suggest that tumor samples with low ATP23 expression are potentially more prone to evading immune responses compared to those with high ATP23 expression. Additionally, analysis of metabolic pathway activities demonstrated consistently enhanced engagement of metabolic pathways—including the TCA cycle, oxidative phosphorylation, and glycolysis—in ATP23-high T cells relative to ATP23-low cells (Fig. 8G). These observations align with our bulk RNA sequencing data. Furthermore, functional analysis demonstrated that ATP23 expression was markedly upregulated during the stages of T cell activation and proliferation, while it remained relatively low during the maturation stage when T cells exerted regulatory, cytotoxic, and helper functions (Figs. S6B–S6C).

### Low ATP23 expression predicts impaired intercellular communication ability

**Figure 9:**
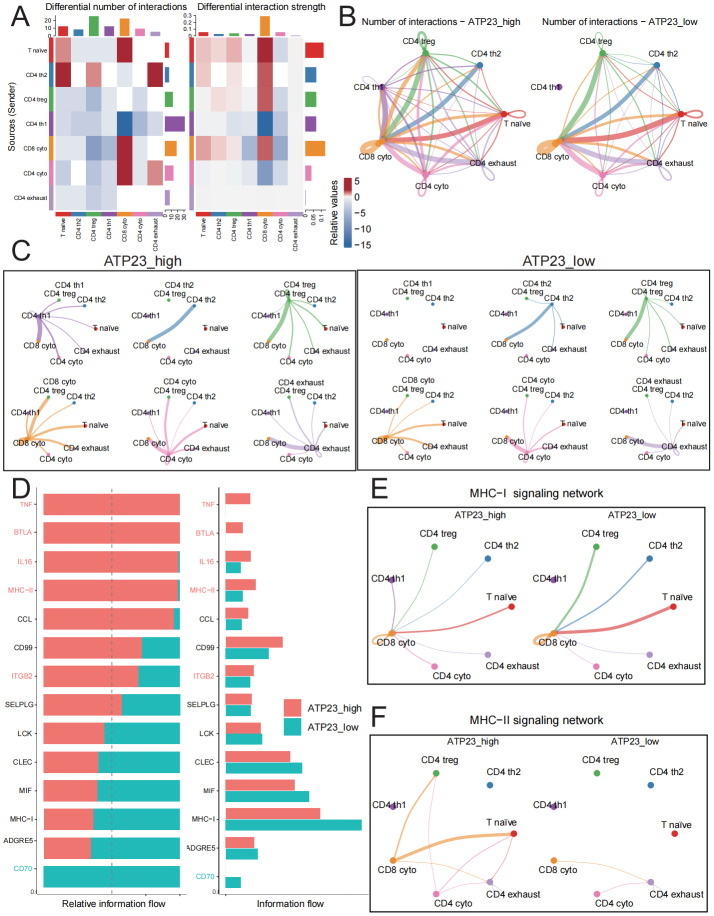
Cell crosstalk between CD8 + and CD4 + T cells was more frequent in the ATP23 high expression group. (A) Analyze the number of interactions and interaction strength among different T cell subsets. The top bar plot represents the summation of column values in the heatmap, which corresponds to incoming signaling, while the right bar plot indicates the summation of row values, representing outgoing signaling. The presence of red or blue in the color bar signifies an increase or decrease signal in signal intensity within the ATP23 low group compared to the ATP23 high group. (B) Comparison of the number of intercellular interactions among different subtypes of T cells between ATP23 high and low groups. The color of the edgescorresponds to the originating cell, whereas the thickness of the edges is proportional to the number of interactions. (C) Ligand–receptor connections between T cell subtypes under ATP23 high (left panel) or low (right panel) conditions. The color of the edgescorresponds to the originating cell, whereas the thickness of the edges signifies the magnitude of interactions. (D) The comparative analysis of the overall information flow in different signaling pathways between the two groups. The red and blue labels indicate that the FDR-adjusted *P*-values for both groups are statistically significant at the 0.05 level. (E–F) Ligand–receptor connections of MHC-I (E) and MHC-II (F) signaling pathways under ATP23 high or low conditions. The color of the edges corresponds to the originating cell, while the thickness of the edges signifies the magnitude of interactions.

The intercellular communication among T cell subpopulations facilitates the enhanced recognition and efficient elimination of tumor cells by cytotoxic T lymphocytes (CTL) (Raskov et al., 2021). For instance, *via* the MHC-II signaling pathway, CD4 th1 cells can induce DC cells to secrete IL-12 or IL-15 in order to augment clonal proliferation of CD8 T cells and promote CTL differentiation (Borst et al., 2018). As described in Fig. 8A, we categorized tumor samples into two groups: the ATP23_low group and the ATP23_high group. Although there was no significant disparity in overall communication intensity between these two groups, the number of cellular communications in the ATP23_low group was comparatively lower than that in the ATP23_high group (Fig. 9A). When combined with Figs. 9B–9C, it became evident that CD8 cyto exhibit an enhanced overall input signal but a reduced output signal capability in the ATP23_low group. In contrast, both CD4 th1 and CD4 treg cells demonstrated diminished overall input and output signal capacities under the same condition. These observations suggest that reduced ATP23 expression may impair T cell energy synthesis, thereby compromising cellular immune function.

The subsequent analysis was directed towards elucidating the divergences in expression patterns of specific signaling pathways between the two groups (Fig. 9D and Table S6). The group with high ATP23 expression exhibited evident activation of multiple immune pathways, including the tumor necrosis factor (TNF) signaling pathway and BTLA signaling pathway, while the CD70 signaling pathway was found to be suppressed in this group. The MHC-I signaling pathway demonstrates heightened activity in the ATP23_low group, wherein various cell types (excluding CD4 th1) transmit signals to CD8 cyto *via* this pathway to enhance their cytotoxicity against tumor cells (Fig. 9E). In contrast, the MHC-II signaling pathway exhibits increased activation in the ATP23_high group, facilitating communication between CD4 cyto and CD8 cyto with other T cell subpopulations for sustained activation and tumor eradication (Fig. 9F). These results suggest that tumors with high ATP23 expression exhibit a heightened intercellular communication and enhanced anti-tumor efficacy.

### ATP23 predicts therapeutic response

**Figure 10:**
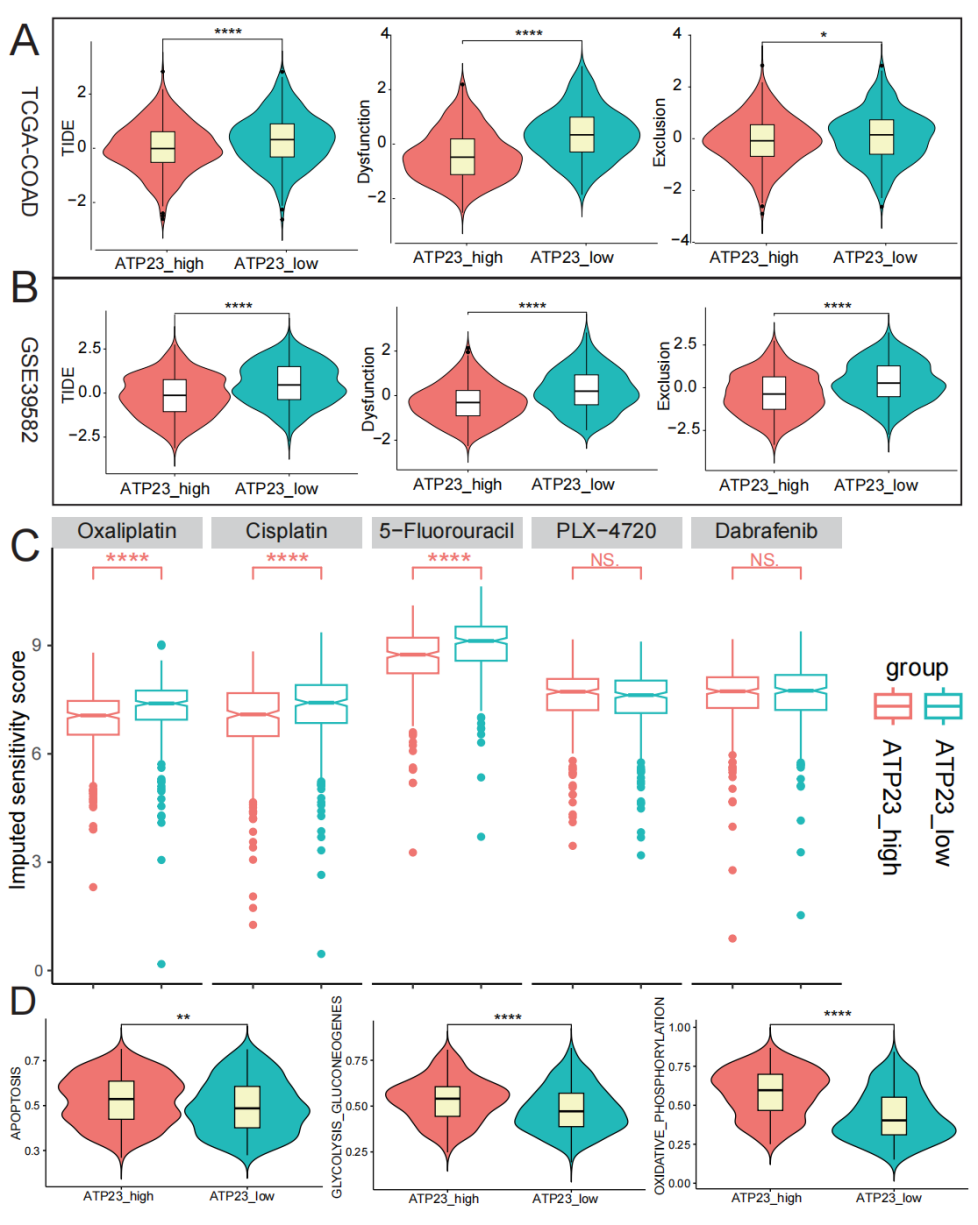
The COAD patients exhibiting high ATP23 expression might potentially exhibit enhanced sensitivity towards immune checkpoint inhibitor (ICI) therapy, as well as platinum-based chemotherapy in combination with 5-fluorouracil. (A–B) Relationship between ATP23 -high/-low group and tumor immune dysfunction and exclusion (TIDE) scores in (A) TCGA-COAD (ATP23_high, *n* = 235; ATP23_low, *n* = 238) and (B) GSE39582 (ATP23_high, *n* = 283; ATP23_low, *n* = 283). (C) The IC50 values of five chemotherapy drugs were compared between high- and low- ATP23 groups utilizing Student’s *t*-test in TCGA-COAD dataset (ATP23_high, *n* = 235; ATP23_low, *n* = 238). (D) The gene set variation analysis (GSVA) of hallmark gene sets in KEGG between high- and low-ATP23 groups (ATP23_high, *n* = 283; ATP23_low, *n* = 283) (NS., no significance, * *p* < 0.05, ** *p* < 0.01, **** *p* < 0.001).

The TIDE online database was utilized to predict the impact of immunotherapy in the high- and low-ATP23 groups, considering the association between ATP23 expression and the immunosuppressive microenvironment. Utilizing the bulk RNA-sequencing datasets (TCGA-COAD and GSE39582), we discovered that low-ATP23 group exhibited significantly higher dysfunction, exclusion, and TIDE scores compared to the high-ATP23 group, as determined by the TIDE algorithm (Figs. 10A–10B and S7A–S7B; detailed data in Table S7). Consequently, patients belonging to the high-ATP23 group may exhibit a more favorable response to immune checkpoint inhibitor (ICI) therapy. Moreover, the association between ATP23 expression and the sensitivity of common chemotherapeutic drugs in patients with COAD was assessed using the TCGA-COAD dataset (Fig. 10C). The in silico predicted IC50 values of each patient for five agents are detailed in Table S8. Patients exhibiting low ATP23 expression demonstrated significantly higher IC50 values for cisplatin, oxaliplatin, and 5-Fluorouracil compared to those with high ATP23 expression (All *P* < 0.05; detailed data in Table S9). In other words, COAD patients with low ATP23 expression may exhibit not only a diminished response rate to ICI treatment but also reduced sensitivity to 5-FU-based chemotherapy. Our GSEA results indicated a dual mechanism associated with high ATP23 expression: On one hand, it bolstered DNA damage repair, an adverse effect that would intrinsically resist platinum-based and 5-fluorouracil chemotherapy (Fig. S7C). On the other hand, it promoted apoptosis and inhibited the MAPK pro-survival pathway, thereby undermining tumor cell viability (Fig. S7D). This net shift toward cell death may partly explain the observed increase in the effectiveness of traditional chemotherapy, yet detailed mechanisms remain to be fully investigated. Since there was no correlation observed between ATP23 expression levels and BRAF mutation in colon cancer, the *in silico*-predicted efficacy of selective BRAF V600E inhibitors (PLX-4720 and dabrafenib) remained unaffected by ATP23 expression levels. Finally, we applied the GSEA and GSVA algorithms to evaluate the relationship between ATP23 expression and energy metabolism pathways and programmed cell death pathways in GSE39582 dataset (Fig. 10D and Fig. S7E). Notably, our findings revealed that the low ATP23 group exhibited significantly diminished scores in apoptosis, glycolysis gluconeogenesis, and oxidative phosphorylation pathways, thereby indicating a compromised immunogenic capacity and impaired energy production.

**Figure 11:**
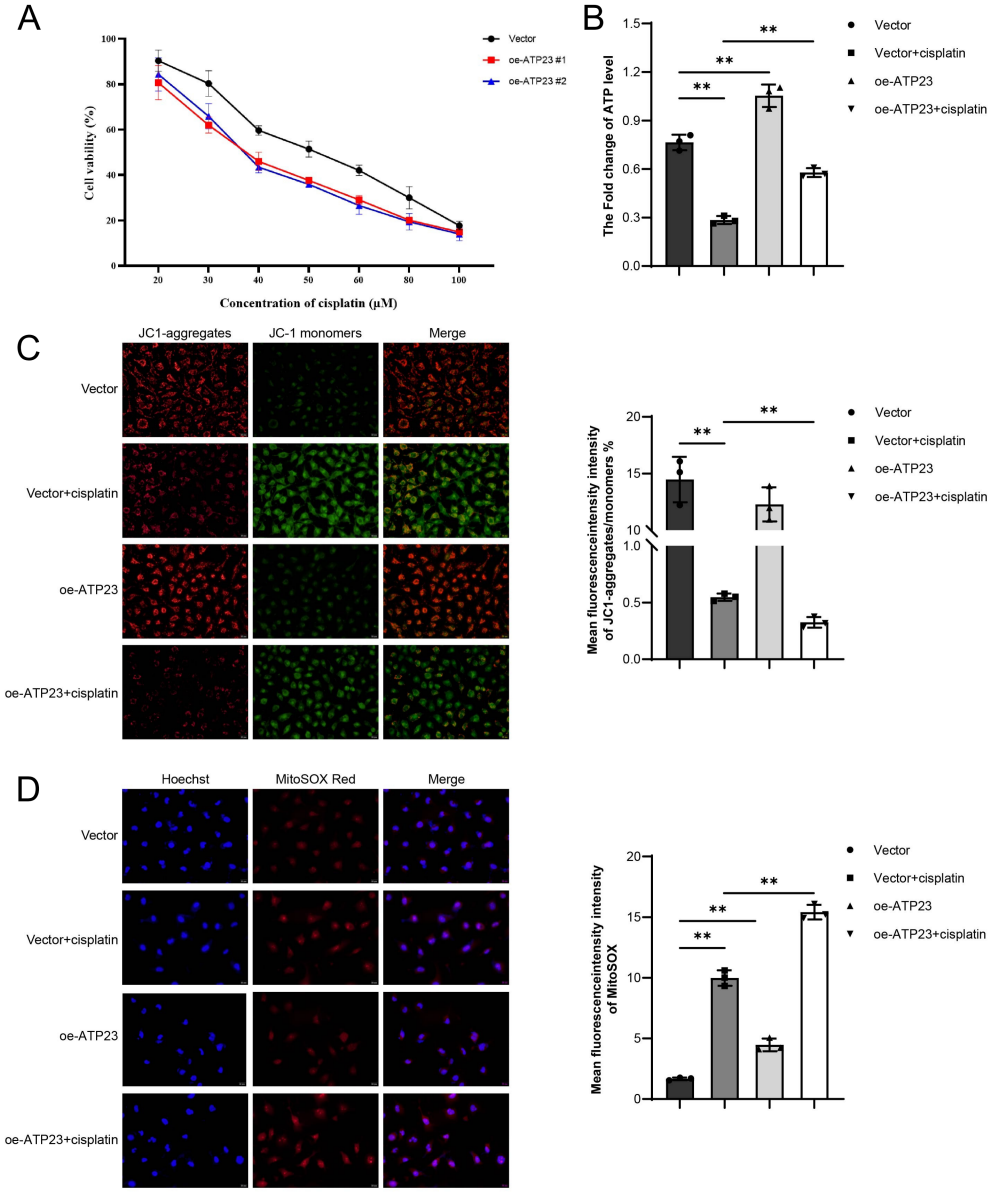
ATP23 overexpression enhances cisplatin sensitivity and promotes oxidative phosphorylation in HCT116 cells. (A) Cisplatin sensitivity was assessed in HCT116 cells transfected with an ATP23 overexpression plasmid or empty vector control. (B) Intracellular ATP levels were measured to evaluate mitochondrial function. (C) Left panel, typical images of JC-1 immunochemistry staining. Scale bar: 20 µm. Right panel, elevated expression of ATP23 mediates cisplatin-induced mitochondrial dysfunction. (D) Left panel, representative images of MitoSOX Red staining. Scale bar: 20 µm. Right panel, upregulation of ATP23 leads to elevated levels of reactive oxygen species (* *p* < 0.05, ** *p* < 0.01, compared with the NC group; *n* = 3).

*In vitro* experiments were conducted to evaluate the effects of ATP23 overexpression on sensitivity to platinum-based chemotherapy and oxidative phosphorylation activity. As shown in Fig. 11A, HCT116 cells overexpressing ATP23 exhibited increased sensitivity to cisplatin toxicity compared to control cells (Vector: IC50 = 56.95 *μ*M; oe-ATP23 #1: IC50 = 42.55 μM; oe-ATP23 #2: IC50 = 42.91 μM). Therefore, subsequent experiments were performed using oe-ATP23 #1 in combination with 40 *μ*M cisplatin. ATP levels in ATP23-overexpressing cells were significantly higher than those in vector control cells. Consistently, the oe-ATP23 + cisplatin group showed markedly elevated intracellular ATP content compared to the vector + cisplatin group (Fig. 11B). A reduced JC-1 aggregate/monomer ratio, weaker red fluorescence, and stronger green fluorescence were observed in the vector + cisplatin group relative to the vector group, indicating mitochondrial depolarization. Notably, the oe-ATP23 + cisplatin group exhibited the lowest JC-1 aggregate/monomer ratio, the weakest red fluorescence, and the most intense green fluorescence, suggesting that ATP23 overexpression exacerbates mitochondrial dysfunction and promotes apoptosis (Fig. 11C). Fluorescence analysis further revealed increased MitoSOX-derived red fluorescence in the oe-ATP23 + cisplatin group compared to the vector + cisplatin group, indicating elevated reactive oxygen species levels upon ATP23 overexpression (Fig. 11D). Collectively, these findings demonstrated that upregulation of ATP23 increased oxidative phosphorylation and contributed to cisplatin-induced mitochondrial dysfunction in COAD.

### The expression patterns of ATP23 vary across different tumor types

**Figure 12:**
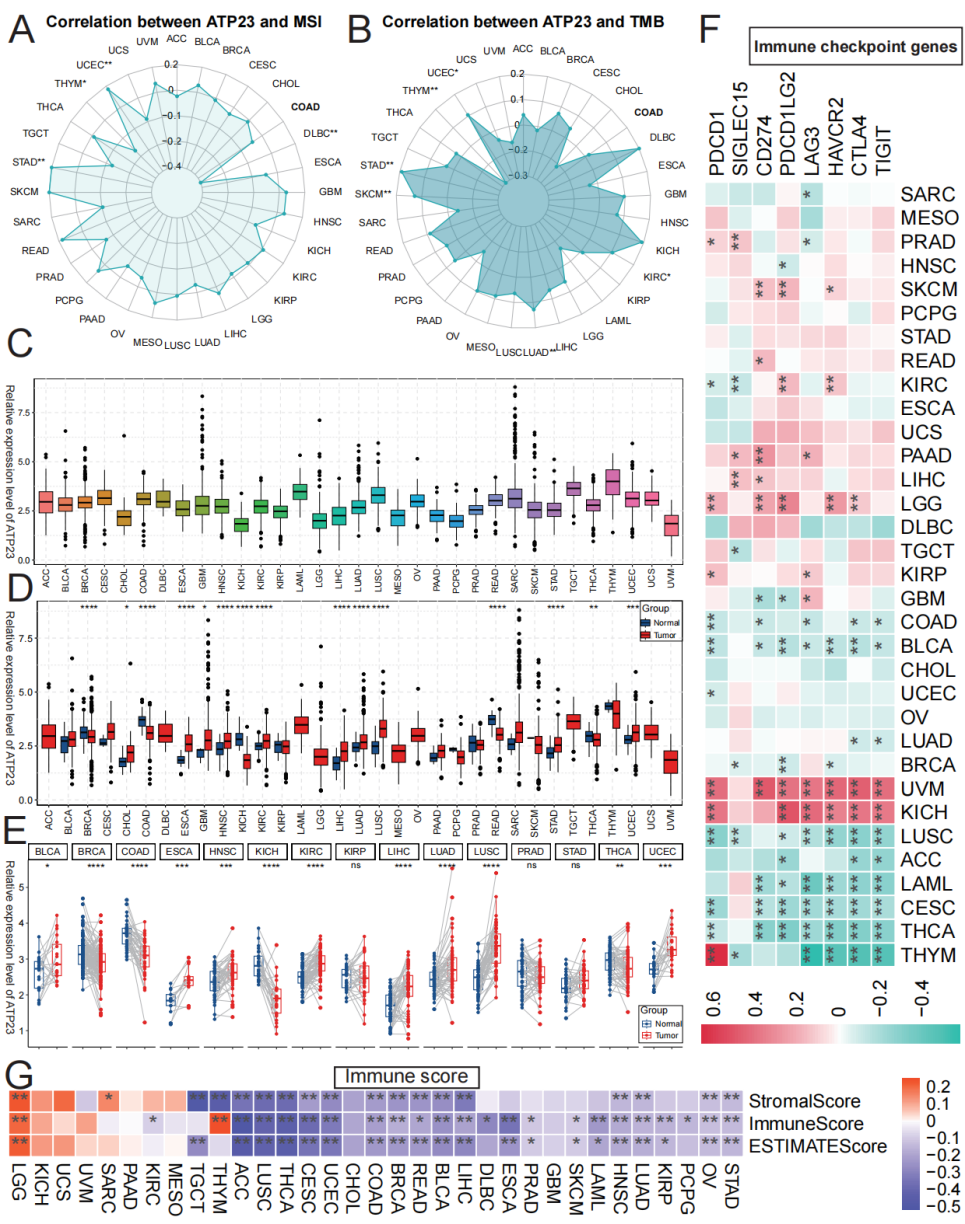
ATP23 pan-cancer analysis. (A–B) The Spearman correlation coefficient was employed to compare the expression of ATP23 with MSI (A) and TMB (B). (C) Relative expression levels of ATP23 in different cancer tissues. (D–E) The relative expression levels of ATP23 in tumor tissues and adjacent non-tumor tissues were compared between unpaired samples (D) and paired samples (E). (F) The correlation between ATP23 expression and immune scores in the TCGA database. (G) The correlation between ATP23 expression and immune checkpoint genes across various tumors (* *p* < 0.05, ** *p* < 0.01, *** *p* < 0.005, **** *p* < 0.001).

We performed a pan-cancer analysis of the expression patterns and immunological role of ATP23 in TCGA database. The results depicted in Figs. 12A–12B indicate that the majority of tumors do not exhibit an association between ATP23 and MSI or TMB. The expression levels of ATP23 show minimal variation across different tumor types, with UVM displaying the lowest levels and THYM exhibiting the highest (Fig. 12C). However, there was significant variation in ATP23 expression levels between multiple tumor tissues and their adjacent normal tissues, both in unpaired and paired samples. In BRCA, COAD, KICH, and THCA, ATP23 was expressed at higher levels in normal tissue compared to tumor tissue; however, the opposite trend was observed in ESCA, HNSC, KIRC, LIHC, LUAD LUSC, and UCEC (Figs. 12D–12E). The relationship between immune checkpoint genes and ATP23 expression exhibited significant heterogeneity across different tumors (Fig. 12F). In the case of COAD, there was an inverse correlation between most immune checkpoint genes and ATP23 expression, whereas in READ, the opposite trend was observed. Regarding stromalScore, immuneScore, and ESTIMATEScore (Fig. 12G), they demonstrated an inverse association with ATP23 expression in the majority of tumors except for LGG (where a significant positive correlation was found). The findings suggest that, similar to COAD, in the majority of tumors, decreased ATP23 levels are associated with increased infiltration of stromal cells and immune cells within the TME. However, as cellular energy production is impaired, increased infiltration of immune cells does not necessarily indicate a more favorable prognosis.

## Discussion

The CMS4 subtype of colon cancer, also known as the mesenchymal type, exhibits a higher prevalence in advanced stages of the disease. It is characterized by the upregulation of genes related to epithelial-to-mesenchymal transition (EMT), higher frequency of somatic copy number alterations, abnormal angiogenesis, and activation of the TGF-β signaling pathway. Consequently, it is associated with an unfavorable prognosis and resistance to therapy (Deng et al., 2021; Guinney et al., 2015). BRAF is one of the most commonly mutated oncogenes in human colon cancer, and tend to be more prevalent in CMS1 (Angerilli et al., 2022). Through analysis of the GSE39582 dataset, we observed that a subset of patients harboring BRAF mutations are classified under the CMS4 subtype, which is associated with a poorer prognosis compared to the other three molecular subtypes. During our investigation into the molecular signature of these patients, we observed a significant downregulation of ATP23 expression which was strongly associated with prognosis.

The ATP23 protein is highly conserved and has homologues in fungi, plants, animals, including humans. These homologues share a similar structure with ten or eleven cysteine residues located at conserved positions throughout the entire sequence (Yang et al., 2021; Yang et al., 2022). Previous research on ATP23 primarily focused on its biological function in yeast, such as its involvement in processing the yeast ATP6 precursor and facilitating the assembly of mitochondrial ATP synthase (Yang et al., 2021). In contrast to the available yeast data, there is a scarcity of studies investigating ATP23 expression in humans, and its role in tumors remains unexplored. Our study revealed that the ATP23 expression in colon cancer was independent of BRAF mutations; however, it exhibited a significant decrease in the CMS4 subtype and was strongly associated with poor prognosis within this specific tumor subgroup. Furthermore, ATP23 expression remained unaffected by microsatellite status and clinicopathological characteristics, aligning consistently with the distinctive features of the CMS4 subtype. *In vitro* experiments indicated that ATP23 inhibits the proliferation, migration, and invasion capabilities of COAD cells. Therefore, we posit that the downregulation of ATP23 expression serves as a contributing factor to the unfavorable prognosis observed in CMS4 subtype tumors.

The ATP23 protein exhibits diffuse distribution across various cell types within tumor tissues. GSEA and GSVA analyses have revealed that ATP23 and its associated genes primarily participate in the intracellular oxidative phosphorylation process. The downregulation of ATP23 in cells will inevitably impair the process of mitochondrial aerobic energy production. By conducting *in vitro* experiments, we verified the impact of ATP23 on the proliferation, migration, and invasion capabilities of tumor epithelial cells. Unlike normally differentiated cells, which predominantly utilize mitochondrial oxidative phosphorylation for energy production, cancer cells primarily depend on aerobic glycolysis as their main source of energy generation. To investigate which cell populations are primarily affected by the low-expression of ATP23 in cellular energy metabolism, we subsequently focused our analysis on tumor infiltrating T lymphocytes, a population critical for anti-tumor responses.

While active T cells (CD4 cyto, CD8 cyto, *etc*.) predominantly rely on glycolysis for energy during their battle against tumor cells, a robust capacity for oxidative phosphorylation can still augment the anti-tumor efficacy (Franco et al., 2020; Hu et al., 2019; Madden & Rathmell, 2021). Moreover, T naive and certain regulatory T cells predominantly depend on oxidative phosphorylation for energy metabolism (Yu et al., 2018); therefore, the reduced ATP23 expression will lead to their functional impairment. From the perspective of T cell intercellular communication, a decrease in ATP23 expression in tumor tissue led to a reduction in the overall number of communications between T naive and other T cell subpopulations, albeit with an increased communication intensity. Moreover, the overall ability of CD4 th1 to communicate with other T cells significantly declined. Although the antigen presentation function mediated by the MHC-I pathway remains unaffected or even slightly enhanced, there was a significant impairment in the immune surveillance ability of the MHC-II pathway in conditions where ATP23 expression was reduced. Notably, the activity of the CD70 signaling pathway among T cell subpopulations is significantly augmented in the presence of low ATP23 expression, which is closely associated with tumorigenesis, tumor progression, and unfavorable prognosis across various cancer types (Buchan, Rogel & Al-Shamkhani, 2018; Riether et al., 2020). To sum up, the low expression of ATP23 hampers the anti-tumor immune function of T cells by diminishing oxidative phosphorylation levels.

Tumor immunotherapy is rapidly advancing as a promising approach for cancer treatment (Ghaedrahmati, Esmaeil & Abbaspour, 2023). Currently, the clinical application of immunotherapy primarily focuses on enhancing the anti-tumor capabilities of T cells, encompassing genetically engineered T cell therapy and immune checkpoint blockade (Met et al., 2019). However, the formation of an immunosuppressive microenvironment in tumor tissues restricts the anti-tumor function of CTL, resulting from the dysregulation of various immunosuppressive cells, immune regulatory factors, and metabolites (Kim et al., 2019; Yuan et al., 2023; Zhang et al., 2023). We discovered that the low ATP23 group had downregulated the expression of AA and MHC molecules but upregulated certain tumor-associated immunosuppressive factors in GSE200997. In the TCGA-COAD dataset, patients exhibiting low ATP23 expression demonstrated significantly diminished scores in the immunogenic cell death pathway and elevated levels of ECM-receptor interaction. Additionally, we assessed the correlation between ATP23 expression and response to immunotherapy by employing the TIDE algorithm. COAD samples in the low ATP23 group exhibited elevated immune dysfunction scores and immune exclusion scores, consequently resulting in a higher TIDE score. This indicates that patients with such tumors are more likely to exhibit an unfavorable therapeutic response to ICI therapy and have a poorer prognosis. Therefore, malignant tumors may employ multiple regulatory mechanisms to establish a robust immunosuppressive microenvironment that facilitates tumor growth, fosters immune evasion, and compromises the effectiveness of immunotherapy. Moreover, the expression level of ATP23 was observed to influence the response of COAD patients towards 5-fluorouracil and platinum drugs; nevertheless, the underlying mechanism remains elusive.

However, as a retrospective study, this study has limitations associated with retrospective data collection. The incorporation of multiple datasets for rigorous validation and the implementation of diverse approaches to mitigate batch effects notwithstanding, it is crucial to acknowledge that sampling bias resulting from tumor genome heterogeneity and cross-platform integration can only be minimized rather than completely eliminated. Another limitation is the absence of clinical sample validation linking ATP23 protein expression to molecular subtyping, prognosis, and immune infiltration in colon cancer, which we intend to pursue as a key objective in follow-up studies. Finally, additional experimental validation using xenograft animal models is imperative to elucidate the role of ATP23-related genes in regulating the efficacy of chemotherapy and immunotherapy.

## Conclusions

The present study conducted a comprehensive analysis of ATP23 expression, highlighting its prognostic significance and correlation with immune response in COAD. Further comprehensive investigations are warranted to elucidate the potential mechanisms underlying the promotion of oxidative phosphorylation in mitochondria. In summary, ATP23 has emerged as a promising prognostic biomarker, while reduced ATP23 expression may inhibit oxidative phosphorylation in T cells and contribute to the formation of an immunosuppressive microenvironment in colon adenocarcinoma.

## Supplemental Information

10.7717/peerj.20838/supp-1 Supplemental Information 1 The representative western blots of three independent experiments for two cell lines

10.7717/peerj.20838/supp-2 Supplemental Information 2 (A–B) The scale free topology fit plot (A) and mean connectivity plot (B) to demonstrate the appropriateness of the chosen power (C) Module eigengene dendrogram in the WGCNA. (D) GS-MM scatter plot showing the analysis of module membership versus gene significance.

10.7717/peerj.20838/supp-3 Supplemental Information 3 (A) The proportional hazards assumption using Schoenfeld residuals (B) Multivariate COX regression analysis identifying prognostic factors of COAD in GSE39582. AJCC, American Joint Committee on Cancer; EOCC, early-onset colon cancer; LOCC, late-onset colon cancer. (C) Calibration slope and intercept for the nomogram. (D) Time-dependent ROC analyses at 36, 60, and 90 months for colon cancer patients in GSE39582.

10.7717/peerj.20838/supp-4 Supplemental Information 4 (A) qRT-PCR analysis of the expression of ATP23 in HCT116 and HT29 cell lines transfected with either an ATP23 overexpression plasmid or an empty vector (B) CCK-8 assays for cell proliferation rates. (C) The scratch wound healing assays for migratory ability. Wound closure areas were quantified relative to the initial wound area using ImageJ software. Scale bar: 200 μm. (D) Colony formation assays for proliferation ability. (E) The mRNA expression of E-cadherin, N-cadherin, and vimentin were detected by qRT-PCR. (* *p* < 0.05, ** *p* < 0.01, compared with the NC group; *n* = 3)

10.7717/peerj.20838/supp-5 Supplemental Information 5 (A) Correlation analysis between immune cell infiltration, adjusted by tumor purity, and ATP23 expression in the GSE17538 dataset (B–C) Association between ATP23 expression and the immune cell subtype infiltration as estimated by the xCell algorithm in the TCGA-COAD dataset (B) and the GSE39582 dataset (C). (ns, no significance, * *p* < 0.05, ** *p* < 0.01, **** *p* < 0.001).

10.7717/peerj.20838/supp-6 Supplemental Information 6 (A) The quality controls flowchart for single-cell RNA sequencing data generation Left panel, n Count, nFeature, and percent.mito metrics in the GSE200997 dataset; Middle panel, nFeature distribution of single and double cells identified by the scDblFinder package; Right panel, proportion of cells removed at each filtering step. (B) Spearman correlation coefficient compared ATP23 expression with T cell activity markers. (C) GSEA for ATP23 correlation with T cell functional gene sets.

10.7717/peerj.20838/supp-7 Supplemental Information 7 (A–B) Scatterplots show linear relationships between ATP2A3 expression and TIDE algorithm-derived scores (TIDE, Dysfunction, Exclusion) in the TCGA-COAD (A) and GSE39582 (B) datasets. (C–D) GSEA for ATP23 correlation with DNA damage repair (DDR) pathways (C) and apoptosis signaling pathways (D). (E) Scatter plots for the linear relationships between ATP23 expression and the gene sets of apoptosis (left panel), glycolysis and gluconeogenesis (middle panel), and oxidative phosphorylation (right panel) derived from the KEGG database.

10.7717/peerj.20838/supp-8 Supplemental Information 8 Statistical data associated with TIDE algorithm

10.7717/peerj.20838/supp-9 Supplemental Information 9 Codebook Translations for the Chinese text in the code repository (https://doi.org/10.5281/zenodo.18163083).

## Additional Information and Declarations

### Data Availability

The following information was supplied regarding data availability: The complete set of raw data is available in the Supplemental Files. The code is available at Zenodo: Li, S. (2026). Anti-oncogenic and immunological functions of ATP23 in CMS4 colon adenocarcinoma based on a machine learning computational framework. Zenodo. https://doi.org/10.5281/zenodo.18163083
