# Supplementary figures and images for: Anti-oncogenic and immunological functions of ATP23 in CMS4 colon adenocarcinoma based on a machine learning computational framework
Source: PeerJ. 2026 Feb 20;14:e20838. doi: 10.7717/peerj.20838 (PMC12927601; doi:10.7717/peerj.20838)

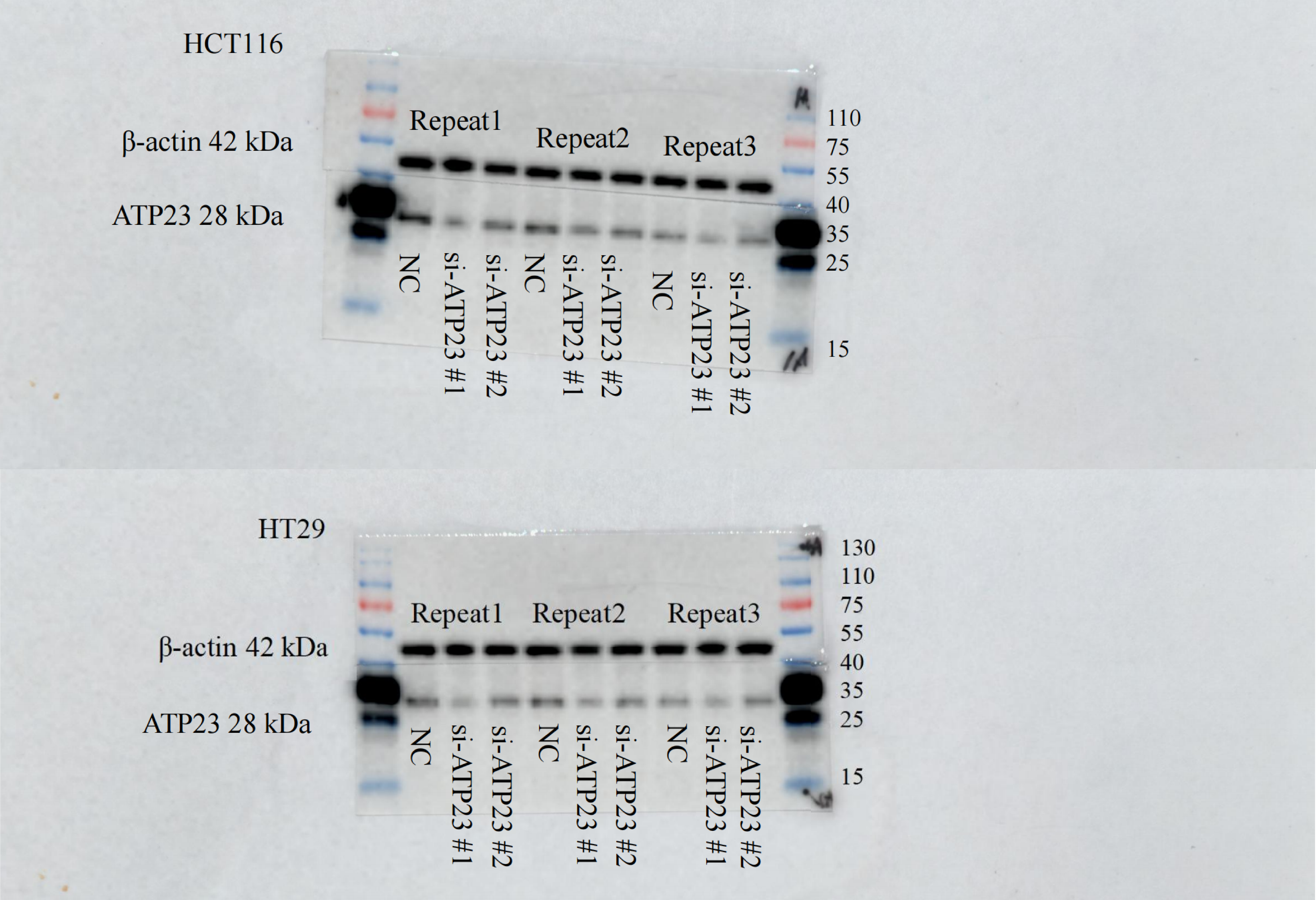

Supplement: Supplemental Information 1 [file peerj-14-20838-s001.png]

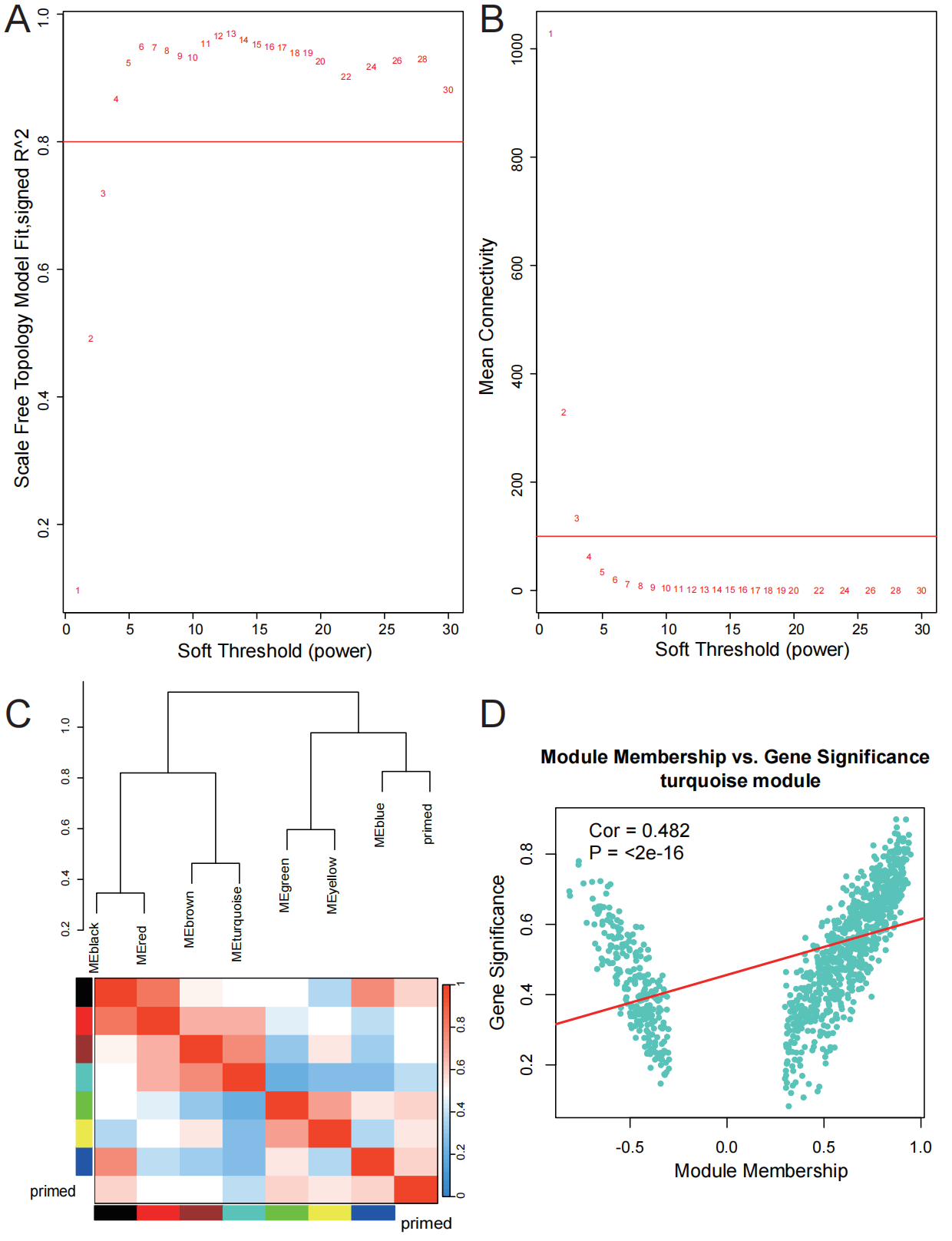

Supplement: Supplemental Information 2 — (C) Module eigengene dendrogram in the WGCNA. (D) GS-MM scatter plot showing the analysis of module membership versus gene significance. [file peerj-14-20838-s002.png]

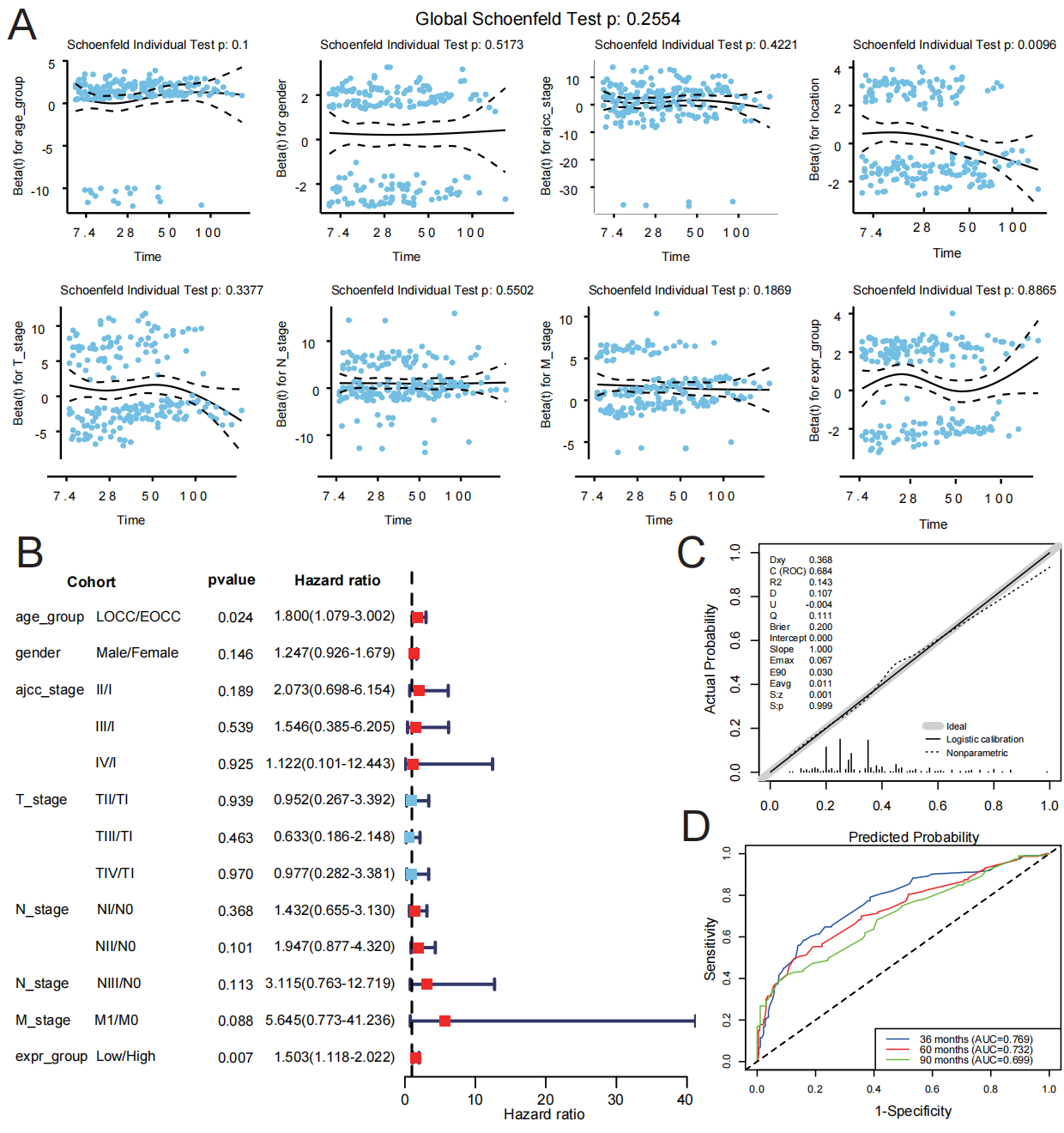

Supplement: Supplemental Information 3 — (B) Multivariate COX regression analysis identifying prognostic factors of COAD in GSE39582. AJCC, American Joint Committee on Cancer; EOCC, early-onset colon cancer; LOCC, late-onset colon cancer. (C) Calibration slope and intercept for the nomogram. (D) Time-dependent ROC analyses at 36, 60, and 90 months for colon cancer patients in GSE39582. [file peerj-14-20838-s003.png]

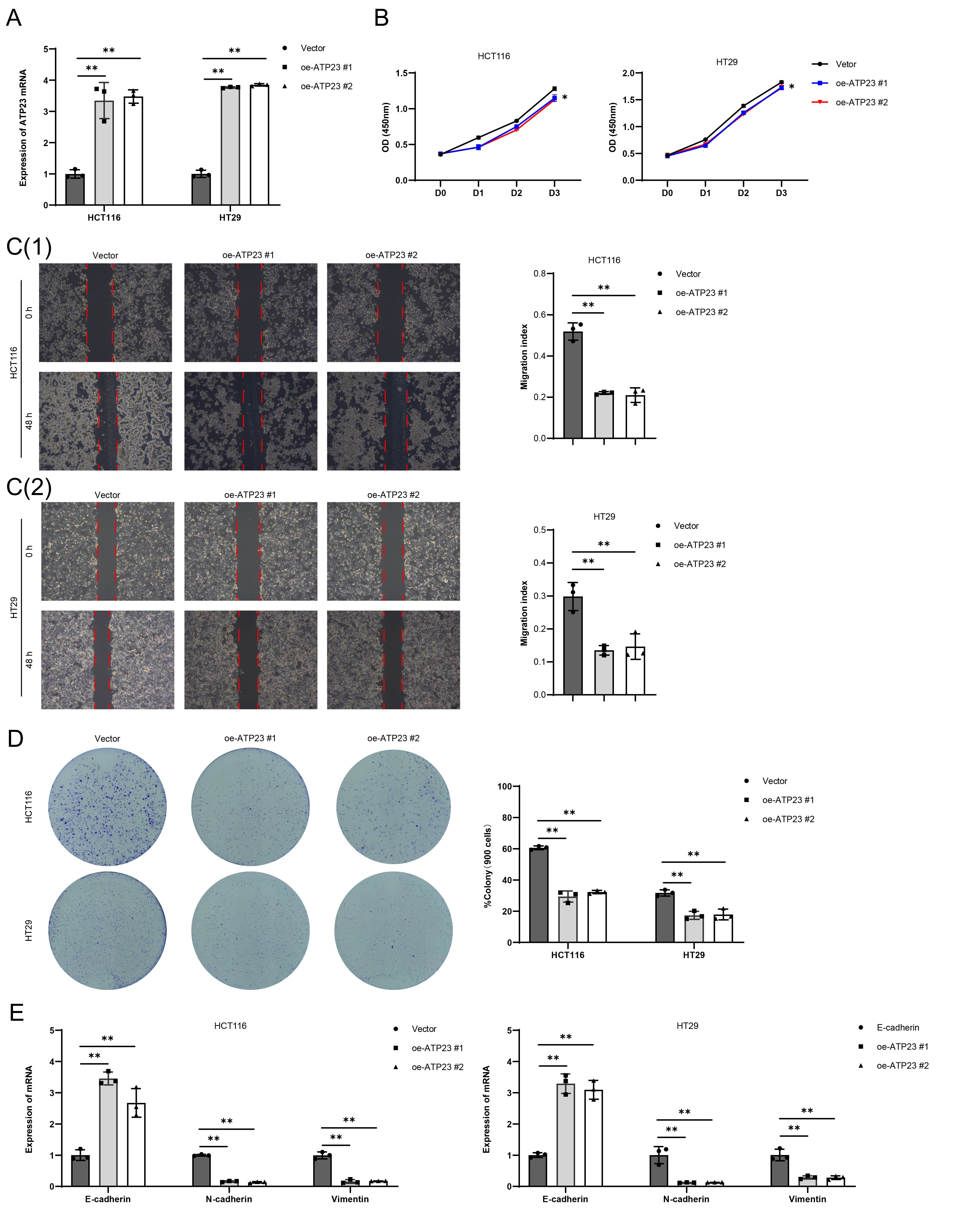

Supplement: Supplemental Information 4 — (B) CCK-8 assays for cell proliferation rates. (C) The scratch wound healing assays for migratory ability. Wound closure areas were quantified relative to the initial wound area using ImageJ software. Scale bar: 200 μm. (D) Colony formation assays for proliferation ability. (E) The mRNA expression of E-cadherin, N-cadherin, and vimentin were detected by qRT-PCR. (* p < 0.05, ** p < 0.01, compared with the NC group; n = 3) [file peerj-14-20838-s004.png]

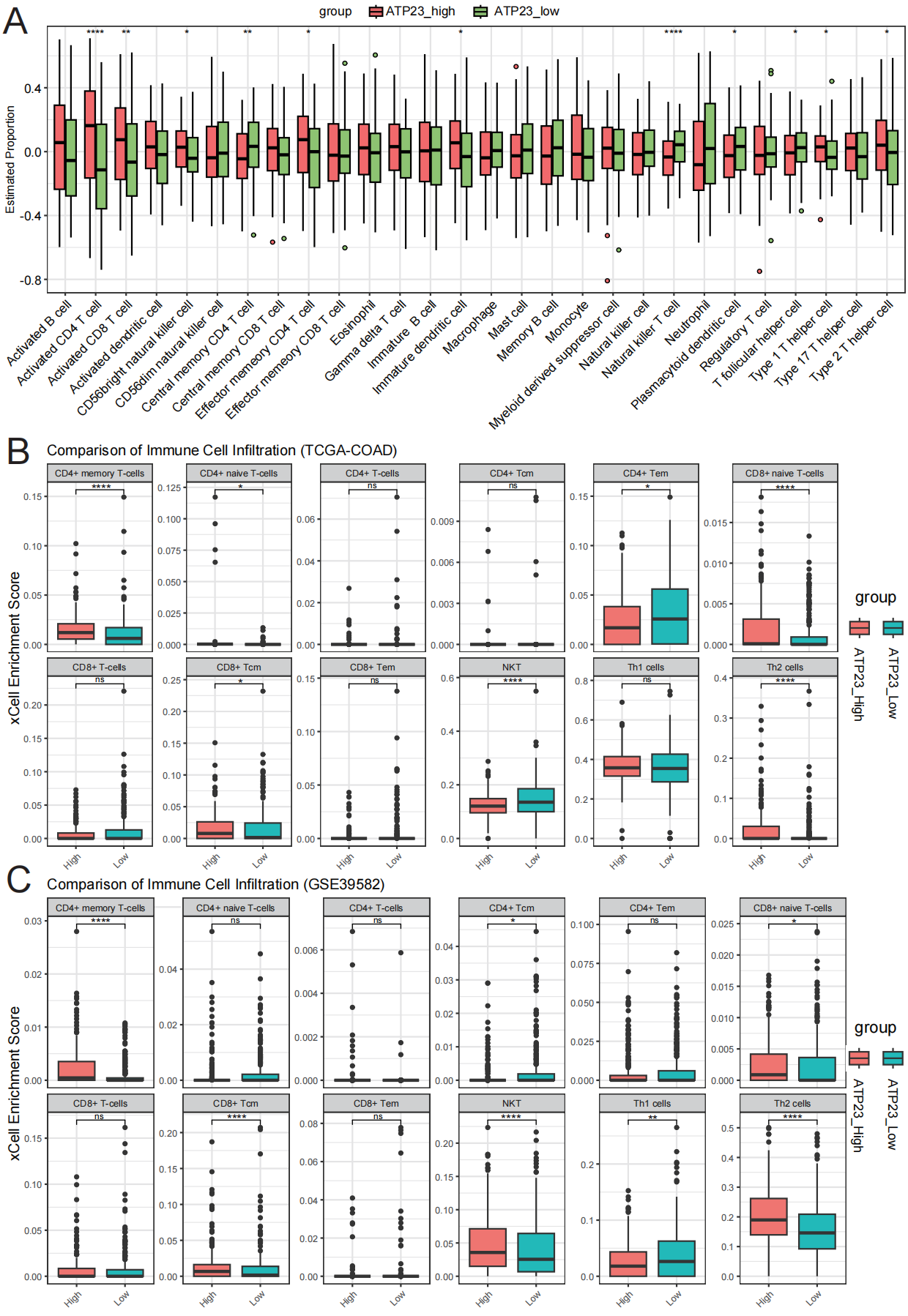

Supplement: Supplemental Information 5 — (B–C) Association between ATP23 expression and the immune cell subtype infiltration as estimated by the xCell algorithm in the TCGA-COAD dataset (B) and the GSE39582 dataset (C). (ns, no significance, * p < 0.05, ** p < 0.01, **** p < 0.001). [file peerj-14-20838-s005.png]

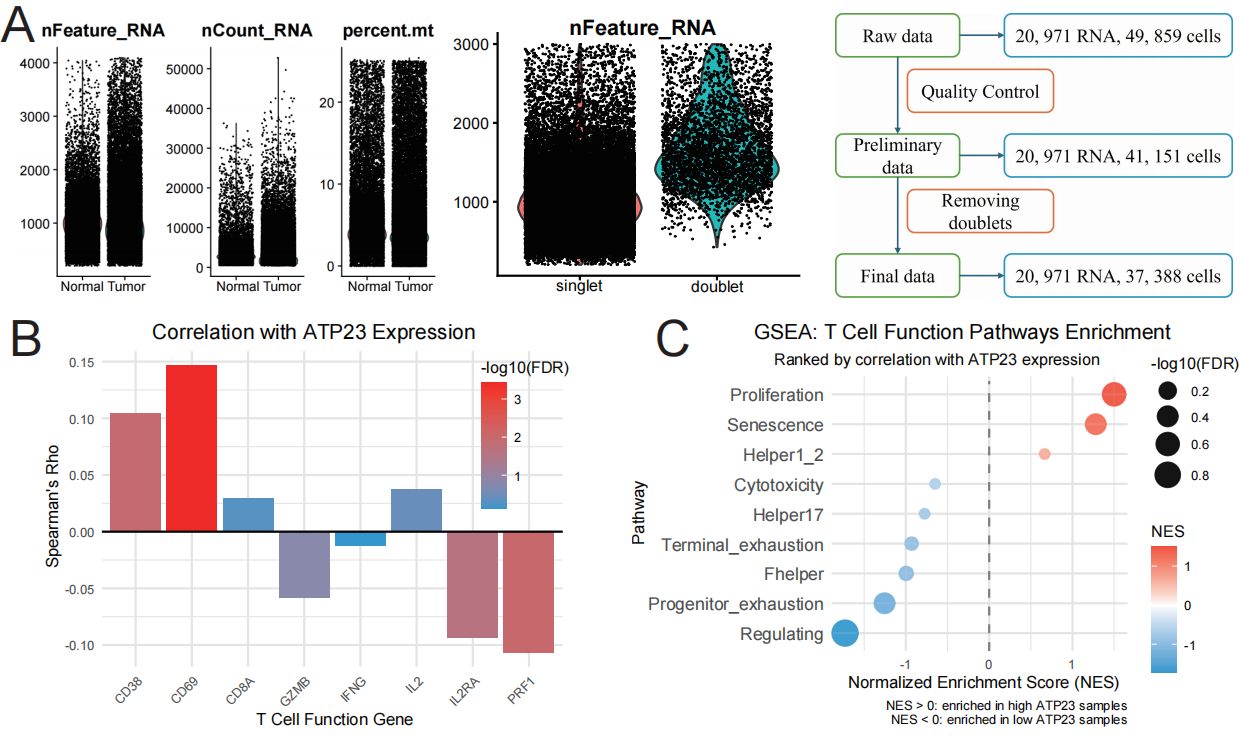

Supplement: Supplemental Information 6 — Left panel, n Count, nFeature, and percent.mito metrics in the GSE200997 dataset; Middle panel, nFeature distribution of single and double cells identified by the scDblFinder package; Right panel, proportion of cells removed at each filtering step. (B) Spearman correlation coefficient compared ATP23 expression with T cell activity markers. (C) GSEA for ATP23 correlation with T cell functional gene sets. [file peerj-14-20838-s006.png]

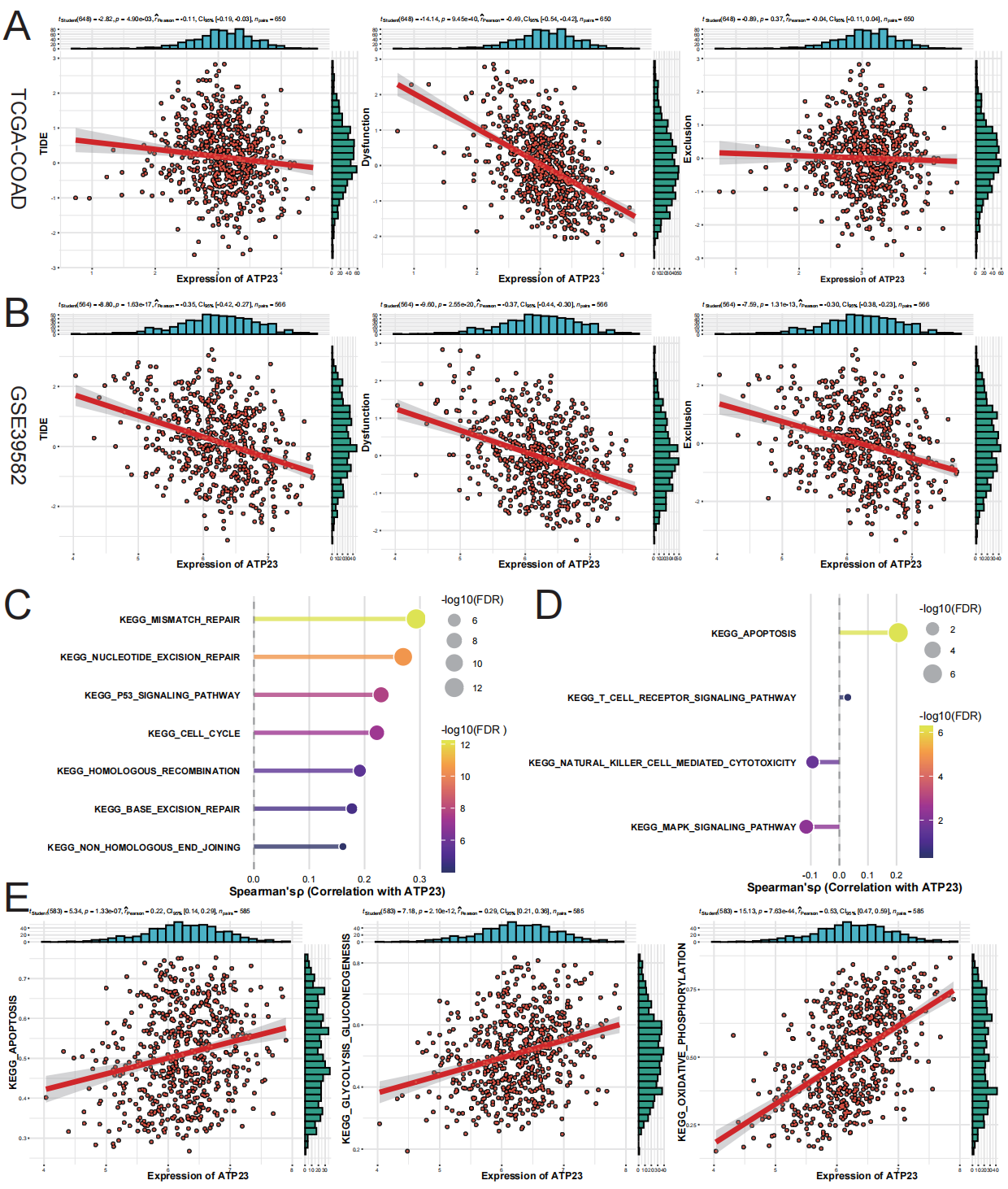

Supplement: Supplemental Information 7 — (A) and GSE39582 (B) datasets. (C–D) GSEA for ATP23 correlation with DNA damage repair (DDR) pathways (C) and apoptosis signaling pathways (D). (E) Scatter plots for the linear relationships between ATP23 expression and the gene sets of apoptosis (left panel), glycolysis and gluconeogenesis (middle panel), and oxidative phosphorylation (right panel) derived from the KEGG database. [file peerj-14-20838-s007.png]
