# Supplementary material for: Anti-oncogenic and immunological functions of ATP23 in CMS4 colon adenocarcinoma based on a machine learning computational framework
Source: PeerJ. 2026 Feb 20;14:e20838. doi: 10.7717/peerj.20838 (PMC12927601; doi:10.7717/peerj.20838)
Supplement: Supplemental Information 9 — Translations for the Chinese text in the code repository (https://doi.org/10.5281/zenodo.18163083). [file peerj-14-20838-s009.docx]

**载入基础包** / Load the foundational R packages.

**WGCNA分析** / WGCNA analysis.

**判断数据质量** / Assess data quality.

**针对样本做聚类树** / Perform cluster analysis on samples.

**若样本有性状、表型，可以添加对应颜色，查看是否聚类合理** / If samples have traits or phenotypes, corresponding colors can be added to check the rationality of clustering.

**绘制样品的系统聚类树及对应性状** / Draw the systematic clustering tree of samples and their corresponding traits.

**判断数据质量：PCA进行分组查看** / Assess data quality: Use PCA for group visualization.

**保存数据** / Save the data.

**挑选最佳阈值power** / Select the optimal soft-thresholding power (beta).

**一步法构建加权共表达网络，识别基因模块** / One-step network construction and module detection for weighted gene co-expression networks.

**模块可视化，层级聚类树展示各个模块** / Visualize modules; display hierarchical clustering dendrogram of modules.

**关联基因模块与表型** / Associate gene modules with phenotypes.

**模块与表型的相关性热图** / Heatmap showing correlations between modules and phenotypes.

**模块与表型的相关性boxplot图** / Boxplot showing correlations between modules and phenotypes.

**批量画boxplot** / Batch generation of boxplots.

**WGCNA可视化** / WGCNA visualization.

**MM vs GS散点图** / Scatter plot of Module Membership (MM) vs. Gene Significance (GS).

**计算模块成员关系** / Calculate module membership relationships.

**创建MM vs GS散点图** / Create scatter plot of MM vs. GS.

**添加拟合线和相关系数** / Add a fitted line and correlation coefficient.

**计算并显示统计量** / Calculate and display statistical metrics.

**机器筛选** / Machine-based screening.

**加载基础包** / Load the foundational R packages.

**构建最优预后模型** / Construct the optimal prognostic model.

**绘制每个模型的 C 指数** / Plot the C-index for each model.

**计算每个模型的AUC分数** / Calculate the AUC score for each model.

**核心基因的选择** / Selection of core genes.

**绘制通过不同方法筛选基因的贡献度** / Plot the contribution of genes screened by different methods.

**根据不同数据集中特定基因的中位表达水平绘制患者生存曲线** / Draw patient survival curves based on the median expression level of specific genes in different datasets.

**癌及癌旁表达可视化** / Visualization of expression in tumor and adjacent normal tissues.

**COAD癌及癌旁** / COAD tumor and adjacent normal.

**GSE44076癌、癌旁及健康对照** / GSE44076 tumor, adjacent normal, and healthy controls.

**TCGA泛癌数据分析** / Pan-cancer analysis using TCGA data.

**ATP23在COAD临床数据相关性** / Correlation of ATP23 with clinical data in COAD.

**基因临床相关分析TCGA** / Gene-clinical correlation analysis in TCGA.

**基因临床相关分析GSE39582** / Gene-clinical correlation analysis in GSE39582.

**ATP23免疫浸润分析** / ATP23 immune infiltration analysis.

**免疫浸润分析（ssGSEA）** / Immune infiltration analysis (ssGSEA).

**细胞纯度矫正** / Correct for cell purity.

**用于偏相关分析** / For partial correlation analysis.

**提取肿瘤纯度列** / Extract the tumor purity column.

**T细胞活性基因** / T-cell activity genes.

**T细胞功能基因集** / T-cell functional gene set.

**TIDE评分** / TIDE score.

**Dysfunction小提琴图** / Violin plot for Dysfunction score.

**Exclusion小提琴图** / Violin plot for Exclusion score.

**效应量计算（重新修改ATP23_GSE39582_TIDE表格，只包含三列数据,TIDE_Group去除ATP23一列）** / Effect size calculation (re-modify the ATP23_GSE39582_TIDE table to contain only three columns of data, remove the ATP23 column from the TIDE_Group).

**散点图** / Scatter plot.

**Exclusion小提琴图** / Violin plot for Exclusion score.

**散点图** / Scatter plot.

**ATP23在GSE39582中富集分析** / Enrichment analysis of ATP23 in GSE39582.

**载入基础包** / Load the foundational R packages.

**绘制图例** / Draw the legend.

**差异分析** / Differential expression analysis.

**载入数据** / Load the data.

**构建实验设计矩阵** / Construct the experimental design matrix.

**构建对比模型，比较两个实验条件下表达数据** / Construct the contrast model to compare expression data between two experimental conditions.

**线性模型拟合** / Fit a linear model.

**根据对比模型进行差值计算** / Perform differential expression calculation based on the contrast model.

**贝叶斯检验** / Empirical Bayes test.

**生成基因的检验结果报告** / Generate a test results report for genes.

**我们使用|logFC| > 0.5，padj < 0.05（矫正后P值）** / We use |logFC| > 0.5 and padj < 0.05 (adjusted P-value).

**筛选出所有差异基因的结果** / Filter to obtain the results for all differentially expressed genes.

**绘制火山图** / Draw a volcano plot.

**绘制热图** / Draw a heatmap.

**按照差异重新排序,挑选前30** / Re-order based on differential expression, select the top 30.

**颜色设置** / Color settings.

**颜色设置** / Color settings.

**GSEA及GSVA分析** / GSEA and GSVA analysis.

**GSEA富集图** / GSEA enrichment plot.

**小提琴图** / Violin plot.

**散点图** / Scatter plot.

**突变分析** / Mutation analysis.

**处理突变数据** / Process mutation data.

**在数据结构中添加上述部分** / Add the above section to the data structure.

**处理临床数据** / Process clinical data.

**瀑布图：高频突变的前10个基因，Multi_Hit的变异是指在同一样本中发生多次突变的基因** / Waterfall plot: Top 10 genes with high-frequency mutations; Multi_Hit refers to genes with multiple mutations in the same sample.

**计算TMB** / Calculate TMB (Tumor Mutational Burden).

**生存分析** / Survival analysis.

**单因素回归分析** / Univariate regression analysis.

**使用 ezcox 进行批量 Cox 模型处理** / Batch processing of Cox models using ezcox.

**处理数据，去掉status缺失值** / Process data, remove samples with missing 'status'.

**单个变量** / Single variable.

**多个变量** / Multiple variables.

**构建tabletext** / Construct table text.

**绘制nomogram，可视化cox比例风险模型的结果** / Draw a nomogram to visualize the results of the Cox proportional hazards model.

**Bootstrap是一种统计方法，它通过从原始数据中有放回地进行重采样来估计参数的不确定性和分布。在这里，Bootstrap用于生成多个随机样本来估计校准曲线的分布，以便获得更可靠的校准结果。** / Bootstrap is a statistical method that estimates parameter uncertainty and distribution by resampling with replacement from the original data. Here, Bootstrap is used to generate multiple random samples to estimate the distribution of the calibration curve, thereby obtaining more reliable calibration results.

**绘制校准曲线** / Draw the calibration curve.

**使用nomogramFormula计算每个患者的列线图得分** / Use nomogramFormula to calculate the nomogram score for each patient.

**多因素回归分析** / Multivariate regression analysis.

**加载必要的库** / Load the necessary libraries.

**数据准备与预处理** / Data preparation and preprocessing.

**将分类变量转换为因子并设置参考水平** / Convert categorical variables to factors and set reference levels.

**使用 lrm() 拟合逻辑回归模型，计算calibration slope and intercept** / Use lrm() to fit a logistic regression model, calculate calibration slope and intercept.

**多因素森林图** / Multivariate forest plot.

**比例风险 (PH) 诊断** / Proportional hazards (PH) assumption diagnostic.

**模型性能：C-index 与乐观校正** / Model performance: C-index and optimism-corrected C-index.

**时间依赖性 ROC 曲线** / Time-dependent ROC curve.

**绘制ROC曲线** / Draw the ROC curve.

**药物治疗** / Drug treatment.

**数据库验证** / Database validation.

**效应量计算（重新修改ATP23_GSE39582_TIDE表格，只包含三列数据）** / Effect size calculation (re-modify the ATP23_GSE39582_TIDE table to contain only three columns of data).

**获取KEGG通路基因集** / Retrieve KEGG pathway gene sets.

**选择我们感兴趣的特定DDR通路** / Select specific DDR pathways of interest.

**DDR相关通路** / DDR-related pathways.

**凋亡相关通路** / Apoptosis-related pathways.

**进行ssGSEA分析** / Perform ssGSEA analysis.

**使用Spearman等级相关** / Use Spearman rank correlation.

**可视化-绘制条形图** / Visualization - Draw a bar plot.

**单细胞测序** / Single-cell RNA Sequencing

**数据集GSE200997前期处理** / Pre-processing of dataset GSE200997.

**质控** / Quality control.

**数据标准化/降维/Harmony去批次** / Data normalization / Dimensionality reduction / Harmony for batch effect correction.

**线性降维（PCA）** / Linear dimensionality reduction (PCA).

**UMAP降维聚类** / UMAP for dimensionality reduction and clustering.

**去除双细胞** / Remove doublets.

**细胞注释** / Cell annotation.

**ggplot修饰单细胞降维聚类图** / Modify single-cell dimensionality reduction clustering plots using ggplot.

**基因表达可视化** / Visualization of gene expression.

**inferCNV画图** / Plotting with inferCNV.

**可视化** / Visualization.

**良恶性细胞ATP23对比** / Comparison of ATP23 expression between benign and malignant cells.

**肿瘤样本热图** / Heatmap for tumor samples.

**T细胞亚群分析** / T-cell subset analysis.

**前期处理** / Pre-processing.

**非线性降维UMAP** / Non-linear dimensionality reduction with UMAP.

**绘图** / Plotting.

**细胞注释** / Cell annotation.

**免疫逃逸** / Immune escape.

**scMetabolism代谢分析** / scMetabolism metabolic analysis.

**注意细胞名对应** / Pay attention to cell name correspondence.

**细胞通讯** / Cell-cell communication.

**创建cellchat对象并进一步处理** / Create a CellChat object and perform further processing.

**计算和推断细胞间通讯网络** / Compute and infer intercellular communication networks.

**合并cellchat对象** / Merge CellChat objects.

**所有细胞群总体观：通讯数量与强度对比** / Overall view for all cell groups: comparison of communication quantity and strength.

**数量与强度差异网络图** / Network diagram showing differences in quantity and strength.

**检查每种细胞发出的信号** / Check signals sent by each cell type.

**通路信号强度对比分析(保守和特异性信号通路的识别与可视化** / Comparative analysis of pathway signal strength (identification and visualization of conserved and specific signaling pathways).

**特定信号通路的对比(MHC-II)为例** / Comparison of specific signaling pathways (using MHC-II as an example).
